# Alternative polyadenylation upon *CPSF6* knock-out enhances HIV-1 infection in primary T cells

**DOI:** 10.1371/journal.ppat.1013745

**Published:** 2025-12-12

**Authors:** Daphne Cornish, Kathryn A. Jackson-Jones, Ted Ling-Hu, Lacy M. Simons, William J. Cisneros, Edmund Osei Kuffour, Natalie Stegman, Francesca Agnes, Yujin Lee, Ved P. Sharma, Paul D. Bieniasz, Ramon Lorenzo-Redondo, Judd F. Hultquist

**Affiliations:** 1 Division of Infectious Diseases, Northwestern University Feinberg School of Medicine, Chicago, Illinois, United States of America; 2 Center for Pathogen Genomics and Microbial Evolution, Northwestern University Havey Institute for Global Health, Chicago, Illinois, United States of America; 3 Laboratory of Retrovirology, The Rockefeller University, New York, New York, United States of America; 4 Bio-Imaging Resource Center, The Rockefeller University, New York, New York, United States of America; 5 Howard Hughes Medical Institute, The Rockefeller University, New York, New York, United States of America; Loyola University Chicago, UNITED STATES OF AMERICA

## Abstract

Human immunodeficiency virus (HIV) relies upon a broad array of host factors in order to replicate and evade the host antiviral response. Cleavage and polyadenylation specificity factor 6 (CPSF6) is one such host factor that is recruited by incoming HIV-1 cores to regulate trafficking, nuclear import, uncoating, and integration site selection. Despite these well-described roles, the impact of CPSF6 perturbation on HIV-1 infectivity varies considerably by cell type. Here, we report that *CPSF6* knock-out in primary CD4+ T cells leads to increased permissivity to HIV-1 infection due to broad transcriptional reprogramming. Knock-out of *CPSF6* results in widespread differential gene expression, including downregulation of genes involved in the innate immune response and enhanced expression of the HIV-1 co-receptors. Accordingly, these cells are less responsive to interferon and express lower levels of antiretroviral restriction factors, including TRIM5α. These transcriptional changes are linked to global shortening of mRNA 3’ untranslated regions (UTRs) through changes in alternative polyadenylation (APA), which are triggered by disruption of the CPSF6-containing Cleavage Factor Im (CFIm) complex. Furthermore, we find that recruitment of CPSF6 by HIV-1 cores is sufficient to perturb CPSF6 function, leading to 3’ UTR shortening and subsequent transcriptional rewiring. These results suggest a model in which HIV-1 transcriptionally reprograms target cells through recruitment of CPSF6 to incoming cores to circumvent the antiviral response and enhance permissivity to infection.

## Author summary

The HIV-1 capsid core plays a critical role in facilitating interactions between the virus and the host cell. A variety of host proteins, metabolites, and small molecule drugs can bind to the capsid lattice to modulate HIV-1 infection. The recent FDA approval of Lenacapavir, a first-in-class capsid inhibitor for use in antiretroviral therapies, has underscored the importance of understanding the dynamics of the capsid core and its interaction with host proteins known as host factors. One such host factor is Cleavage and Polyadenylation Specificity Factor 6 (CPSF6), which is recruited by the HIV-1 capsid core. We found that *CPSF6* knock-out primary CD4+ T cells are highly permissive to HIV-1 infection. This permissivity is triggered by disruption of the CPSF6-containing Cleavage Factor Im (CFIm) complex, which regulates cellular gene expression through polyadenylation site selection. *CPSF6* knock-out cells undergo transcriptional changes that enhance infectivity of some viral strains through enhanced co-receptor expression and decreased innate immune function. Furthermore, we find that recruitment of CPSF6 by HIV-1 cores perturbs CFIm function and triggers transcriptional rewiring. Our results uncover a novel role of CPSF6 in HIV-1 replication, which will inform studies of the HIV-1 early lifecycle and the development of next-generation capsid inhibitors.

## Introduction

The HIV-1 core is a metastable structure of capsid hexamers and pentamers that form a fullerene cone encapsulating two copies of the viral RNA genome [1]. After deposition into the host cell cytoplasm, the core shields the viral genome from innate immune sensing while facilitating reverse transcription and trafficking into the nucleus, whereupon it must disassemble to enable integration in a process known as uncoating. These functions are facilitated and counteracted by a number of host factors that bind to the core and regulate its form and function, including cyclophilin A (CYPA) [2–4], tripartite motif-containing protein 5a (TRIM5α) [5,6], nucleoporin 153 (NUP153) [7], and cleavage and polyadenylation specificity factor 6 (CPSF6).

CPSF6 binds to incoming cores in the target cell and has a variety of proposed functions in HIV-1 replication, including in regulation of core trafficking to the nucleus [8], nuclear import [9,10], uncoating [11], and integration site selection [12–18]. A growing body of literature suggests that the primary role of CPSF6 in the HIV-1 lifecycle is in directing HIV-1 localization following entry into the nucleus [12] and in subsequent integration site selection [16]. Additionally, CPSF6 has been proposed to promote immune evasion by shielding viral pathogen associated molecular patterns (PAMPs) from detection [19]. Despite these many roles in the viral lifecycle, reported HIV-1 infection phenotypes upon CPSF6 depletion vary widely by viral strain and cell type, including some conditions that result in an increase in viral infectivity [16,20]. Likewise, HIV-1 capsid mutants that can no longer bind CPSF6 (such as A77V and N74D) often yield conflicting and/or cell-type dependent phenotypes that may suggest pleiotropic effects [21–23].

CPSF6 is a member of the cleavage factor Im (CFIm) complex alongside its binding partner, CPSF5 [24]. The CFIm complex is a major regulator of polyadenylation site selection during host messenger RNA (mRNA) processing [25–27]. Disruption of CFIm function alters post-transcriptional mRNA regulation and triggers changes in alternative polyadenylation (APA), leading to a preference for proximal poly(A) sites and a global shortening of 3’ untranslated regions (UTRs) [28–31]. Changes in APA have several functional consequences, including altered transcript abundance, localization, and translational potential [29]. These changes have been linked with regulation of several cellular pathways, including the antiviral innate immune response [32]. It is unknown if changes to APA can also influence the innate immune response to HIV-1 infection and if this phenomenon is related to the cell-type specific phenotypes observed upon CPSF6 depletion.

To better understand the role of CPSF6 in HIV-1 infection, we used CRISPR-Cas9 gene editing to knock-out *CPSF6* in primary CD4+ T cells. These cells were markedly more permissive to HIV-1 infection in a strain-specific manner. *CPSF6* knock-out resulted in global changes in APA, which correlated with decreased expression of innate immune response genes and increased expression of T cell activation markers and the HIV-1 co-receptors. These cells were less responsive to type I interferon and had decreased expression of known antiretroviral restriction factors. Furthermore, HIV-1 challenge was sufficient to perturb CPSF6 function and induce changes in APA in some cell types in a manner dependent on the capsid-CPSF6 interaction. These studies suggest a new putative model in which normal CPSF6 function can be disrupted by its recruitment to incoming HIV-1 cores, enhancing permissivity to infection by transcriptional reprogramming in a novel form of host shutoff.

## Results

### *CPSF6* knock-out primary CD4+ T cells are more permissive to HIV-1 infection

**Fig 1:**
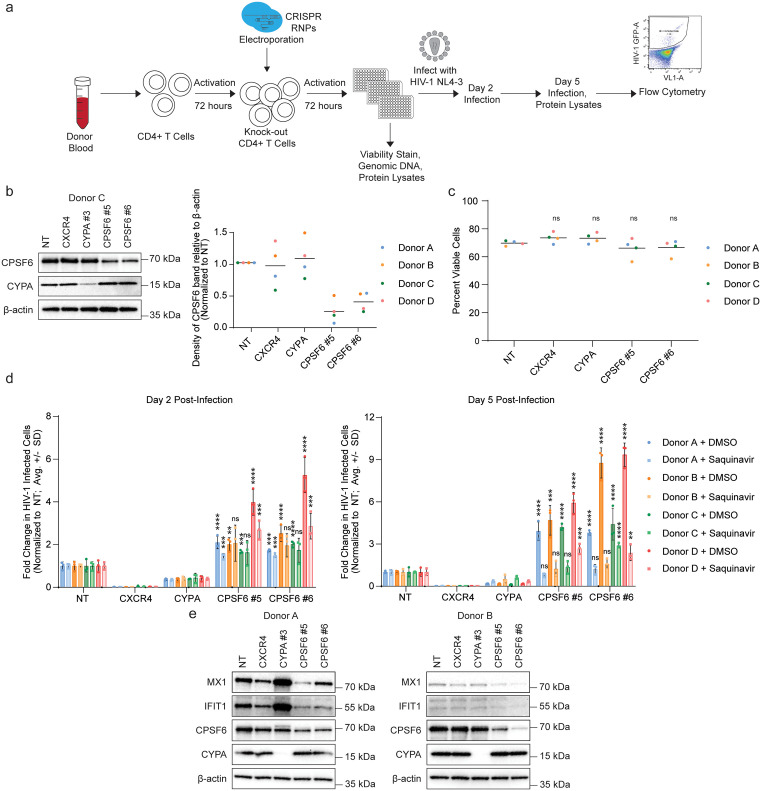
*CPSF6* knock-out in primary CD4+ T cells leads to increased HIV-1 infection rates and dampened innate immune induction. **a,** Experimental workflow for CRISPR-Cas9 editing of primary CD4+ T cells and downstream assays. CD4+ T cells were isolated from human blood, activated, and edited via electroporation of crRNPs. Edited cells were activated, expanded, and split into replica plates for knock-out validation, viability stain, and spreading infection assays. For spreading infection assays, cells were infected with HIV-1 NL4-3 nef:IRES:GFP in technical triplicate per condition and harvested for analysis of infection rates by flow cytometry at days 2 and 5 post-infection. **b,** Immunoblot (left) shows knock-out of *CPSF6* and *CYPA* in primary CD4+ T cell protein lysates harvested at day 4 post-editing in one representative biological replicate (donor C). Chart (right) shows CPSF6 band density divided by β-actin band density in blots from Figs 1b and S1a in 4 biological replicates (donors A-D). Density measurements are normalized to NT controls per donor, horizontal line shows average of biological replicates. **c,** Knock-out primary CD4+ T cells exhibit similar viability to NT controls at day 4 post-editing as assessed by amine dye stain and flow cytometry. Dots represent cell viability (% Ghost Red negative cells) per condition, horizontal lines represent the average of viability measurements in 4 biological replicates (donors A-D). Statistics were calculated relative to the NT control by two-way ANOVA with Dunnet’s test for multiple comparisons; ns = not significant. **d,** HIV-1 infectivity (% GFP positive cells normalized to NT control per donor) at days 2 (left) and 5 (right) post-challenge with HIV-1 NL4-3 nef:IRES:GFP in indicated knock-out primary CD4+ T cells from 4 biological replicates (donors A-D) as assessed by flow cytometry. Cells were treated with protease inhibitor (saquinavir) or DMSO control at 24 hours post-challenge as indicated. Each bar represents the average of technical triplicates + /- SD with individual data points shown. Statistics were calculated relative to the NT control per condition by one-way ANOVA with Dunnet’s test for multiple comparisons; * = p ≤ 0.05, ** = p ≤ 0.01, *** = p ≤ 0.001, **** = p ≤ 0.0001. **e,** Immunoblot showing expression of representative ISGs (*IFIT1* and *MX1*) in protein lysates harvested from HIV-1-infected primary CD4+ T cells at day 5 post-infection in 2 biological replicates (donors A and B).

To better understand the role of CPSF6 in HIV-1 infection, we used CRISPR-Cas9 ribonucleoproteins (crRNPs) to knock-out *CPSF6* in primary CD4+ T cells from 4 independent donors (Fig 1a). Briefly, bulk CD4+ T cells isolated from the peripheral blood of seronegative human donors were activated and electroporated with crRNPs targeting our genes of interest, resulting in polyclonal pools of knock-out cells. Two independent guide RNA targeting *CPSF6* were used in each experiment alongside a non-targeting (NT) negative control guide and two previously validated [3] positive control guides targeting the early-acting host dependency factors *CXCR4* and *CYPA*. Efficient knock-out of *CYPA* and *CPSF6* in each donor was validated by immunoblotting, and quantification of the CPSF6 bands showed substantial reduction in CPSF6 protein levels across all 4 donors (Figs 1b and S1a). *CPSF6* depletion persisted in the polyclonal knock-out cell pool over the course of the experiment (S1b Fig). There was no significant difference in percent viable cells between the NT control and any of the knock-outs as measured by amine dye staining and flow cytometry 4 days post-electroporation (Fig 1c). Daily monitoring of viable cell count by flow cytometry and/or by a luminescent ATP detection assay in 3 additional donors likewise revealed only modest differences in cell proliferation or ATP levels over time, with the *CPSF6* knock-out cells showing only slight decreases in each metric by 10 days post-electroporation (S1c Fig).

To determine the impact of *CPSF6* knock-out on HIV-1 infection, cells were subsequently challenged with cell-free, replication-competent HIV-1 NL4-3 nef:IRES:GFP at 4 days post-electroporation in 6 technical replicates. The following day, 3 replicates were treated with the protease inhibitor Saquinavir (SQV, final concentration 5 µM) to limit infection to a single round of replication, while the other 3 replicates were treated with a matched volume of DMSO as a vehicle control. Percent infected (GFP+) cells were monitored by flow cytometry at 2 and 5 days post-infection and normalized to the NT control (Figs 1d, raw infectivity data S1d). NT cells reached an infection rate of ~1% by day 5 post-infection. As expected, *CXCR4* and *CYPA* knock-out strongly inhibited infection at days 2 and 5, including in the SQV-treated samples, confirming their role in the early phase of the lifecycle. *CPSF6* knock-out, on the other hand, resulted in a 2- to 4-fold increase in infection at day 2, and a 3- to 9-fold increase in infection at day 5. We did not observe a change in GFP mean fluorescence intensity, suggesting that the effect is not being driven by a change in the GFP transcript itself. In the presence of SQV, *CPSF6* knock-out cells maintained a 1.5- to 3-fold increase in infection at both days 2 and 5, suggesting that this increased permissivity is due, at least in part, to changes in the early stage of the lifecycle.

However, unlike knock-out of the *CXCR4* or *CYPA* dependency factors, which resulted in a consistent drop in infection over a 40-fold range of input virus, the effect of *CPSF6* knock-out was dose dependent (S1e Fig). Lower doses of virus resulted in the largest fold changes in infection relative to NT at day 5 while the highest dose challenges resulted in no significant difference. As more virus is added and more cells are infected in the NT condition, the denominator of our normalized fold change calculation increases, which impacts the maximum fold change we can observe. The *CPSF6* knock-out infection rate can only increase until it starts to reach the maximum infection rate achievable in the model. Thus, as we increase the NT infection rate, the overall fold change will eventually approach 1 (S1e Fig). This suggests that *CPSF6* knock-out lowers a surmountable barrier to infection as opposed to facilitating a rate-limiting step that cannot be overcome by mass action.

Upon induction of the innate immune response, interferon (IFN) signaling results in the expression of a myriad of IFN-stimulated genes (ISGs), many of which have antiviral functions [33]. To determine if *CPSF6* knock-out could be altering the innate immune response to infection, we assessed expression of two representative ISGs, IFIT1 and MX1, in primary CD4+ T cells from 2 of the above donors at 5 days post-infection (Fig 1e). IFIT1 and MX1 expression varied by donor in the NT cells. In the donor with higher basal levels of ISGs, *CXCR4* knock-out decreased expression and *CYPA* knock-out increased expression of both ISGs; neither knock-out influenced ISG expression in the donor with lower basal levels. On the contrary, *CPSF6* knock-out cells had decreased expression of both ISGs in both donors compared to the NT control. Thus, while there are some donor-to-donor differences, these results demonstrate that *CPSF6* knock-out enhances HIV-1 infectivity and downregulates ISG induction in primary CD4+ T cells.

### *CPSF6* knock-out primary CD4+ T cells exhibit broad transcriptional reprogramming

**Fig 2:**
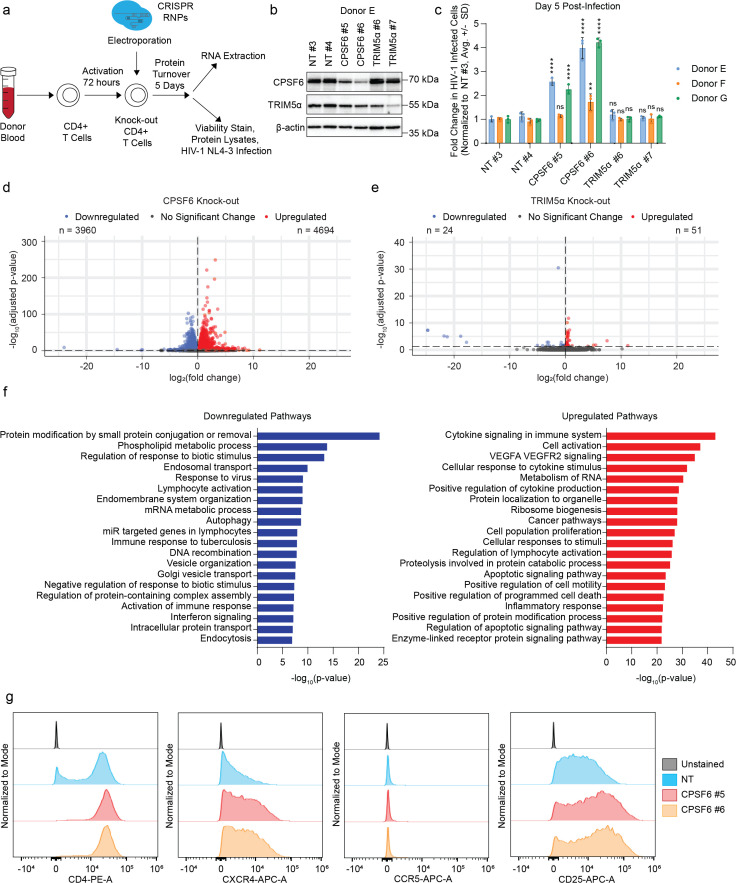
*CPSF6* knock-out primary CD4+ T cells exhibit broad transcriptional reprogramming. a, Schematic shows experimental workflow for RNA-Seq. Primary CD4+ T cells were isolated from the blood of 3 independent donors and edited via electroporation of 2 crRNPs targeting CPSF6 and *TRIM5α* alongside NT controls. RNA was extracted at day 5 post-electroporation and used for bulk RNA-Seq. In parallel, additional cells were used for viability assays, protein knock-out verification, and spreading infection assays. b, Immunoblot shows knock-out of *CPSF6* and *TRIM5α* in primary CD4+ T cell protein lysates harvested at day 5 post-editing in one representative biological replicate (donor E). c, HIV-1 infectivity (% GFP positive cells normalized to NT control per donor) at day 5 post-challenge with HIV-1 NL4-3 nef:IRES:GFP in indicated knock-out primary CD4+ T cells from 3 biological replicates (donors E-G) as assessed by flow cytometry. Each bar represents the average of technical triplicates + /- SD with individual data points shown. Statistics were calculated relative to the NT control per condition by one-way ANOVA with Dunnet’s test for multiple comparisons; ns = not significant, * = p ≤ 0.05, ** = p ≤ 0.01, *** = p ≤ 0.001, **** = p ≤ 0.0001. d, Volcano plot shows differentially expressed genes in RNA-Seq data from uninfected *CPSF6* knock-out primary CD4+ T cells as compared to NT controls in 3 biological replicates (donors E-G). Dots show significantly downregulated (blue) and upregulated (red) genes, dotted line shows threshold of 0.05 for adjusted p-value. e, Volcano plot shows differentially expressed genes in RNA-Seq data from uninfected *TRIM5α* knock-out primary CD4+ T cells as compared to NT controls in 3 biological replicates (donors E-G). Dots show significantly downregulated (blue) and upregulated (red) genes, dotted line shows threshold of 0.05 for adjusted p-value. f, Charts show functional enrichment (Metascape) analysis of top 20 downregulated (left, blue) and upregulated (right, red) pathways in differentially expressed genes from *CPSF6* knock-out primary CD4+ T cells as compared to NT controls in 3 biological replicates (donors E-G). g, Histograms show fluorescence intensity of CD4 (first), CXCR4 (second), CCR5 (third), and CD25 (fourth) in primary CD4+ T cells from 1 representative biological replicate (donor I) at day 5 post-editing as measured by immunostaining and flow cytometry. Y-axis is normalized to the mode, histogram visualized using FlowJo version 10.10.

Given the observed downregulation of ISGs and the known role of CPSF6 in post-transcriptional mRNA processing, we hypothesized that *CPSF6* knock-out could be altering innate immune induction and viral infectivity through transcriptional reprogramming. To explore this hypothesis, we performed bulk RNA-sequencing (RNA-Seq) on uninfected *CPSF6* knock-out primary CD4+ T cells from 3 independent donors (Fig 2a). *CPSF6* knock-out primary CD4+ T cells were generated via CRISPR-Cas9 gene editing as above alongside NT and *TRIM5α* knock-out controls (2 independent guide RNA per condition). TRIM5α is an HIV-1 restriction factor that inhibits the early stage of the viral lifecycle through binding and premature disassembly of incoming cores [5,6]. It has been previously implicated in innate immune signaling upon core binding and oligomerization [34,35]. After allowing 5 days for protein turnover, RNA was extracted from the edited cells and processed for RNA-Seq. *CPSF6* and *TRIM5α* knock-out were confirmed in each donor by immunoblot (Figs 2b and S2a), and each cell population retained high viability (S2b Fig). These *CPSF6* knock-out cells exhibited enhanced permissivity to HIV-1 infection, in line with our previous observations (Fig 1d), while knock-out of *TRIM5α* had no impact on wild-type infection as we previously reported [3] (Fig 2c).

Principal component analysis of the RNA-Seq data revealed that while the NT and *TRIM5α* knock-out specimens clustered tightly together, the *CPSF6* knock-out specimens clustered separately (S2c Fig). After normalization to account for donor-to-donor variation, both guides from each donor were treated as replicates (n = 6 replicates per condition). Differential gene expression analysis revealed broad transcriptional reprogramming of the *CPSF6* knock-out cells as compared to NT controls with 4,694 upregulated and 3,960 downregulated genes (Figs 2d and S2d). In contrast, the transcriptional profile of the *TRIM5α* knock-out cells remained nearly unchanged, with only 51 upregulated and 24 downregulated genes (Figs 2e and S2d).

Functional enrichment analysis revealed that several genes involved in cytokine signaling and T cell activation were upregulated in the *CPSF6* knock-out cells (Fig 2f), including *CD25* and *CXCR4.* To determine if these transcriptional changes were reflected in changes to cell surface expression, we repeated our knock-outs of *CPSF6* and *TRIM5α* using 2 independent guides in 3 additional donors alongside NT, *CXCR4*-targeting, and *CYPA*-targeting controls. At 5 days post-editing, we performed cell surface immunostaining for the HIV-1 receptor CD4, co-receptors CXCR4 and CCR5, and activation marker CD25, quantifying mean fluorescence intensity (MFI) by flow cytometry. While *TRIM5α* knock-out had no significant impact on surface receptor expression, *CPSF6* knock-out cells displayed a slight, but significant increase in the cell surface expression of CD4 and larger increases in cell surface expression of CXCR4 and CD25 (Figs 2g and S2h). *CPSF6* knock-out did not have a significant impact on cell surface expression of CCR5 (Figs 2g and S2h). Thus, increased co-receptor expression and enhanced T cell activation may contribute to the enhanced HIV-1 replication observed in the *CPSF6* knock-out primary CD4+ T cells.

Several pathways involved in the innate immune response were simultaneously observed to be downregulated in *CPSF6* knock-out cells, including response to virus and interferon signaling (Fig 2f). Key components of the type I and type II interferon signaling pathway were identified as being downregulated in each donor, including *IFNAR2*, *STAT3*, *IRF9*, and *JAK2*, while negative regulators of the pathway were upregulated, including *SOCS1* and *SOCS*3 (S2e Fig and S2f). Several ISGs and known antiretroviral restriction factors were also downregulated, including *SAMHD1* and *TRIM5α* (S2e and S2f Fig). Indeed, decreased TRIM5α protein levels were observed in all 3 donors upon *CPSF6* knock-out in the knock-out validation immunoblots (Figs 2b and S2a, quantification in S2g). These results suggest that *CPSF6* knock-out leads to broad transcriptional reprogramming in primary CD4+ T cells that results in upregulation of the viral receptors and downregulation of the innate immune response, which ultimately yields increased permissivity to HIV-1 infection.

### HIV-1 infection phenotypes in *CPSF6* knock-out cells vary by viral strain

**Fig 3:**
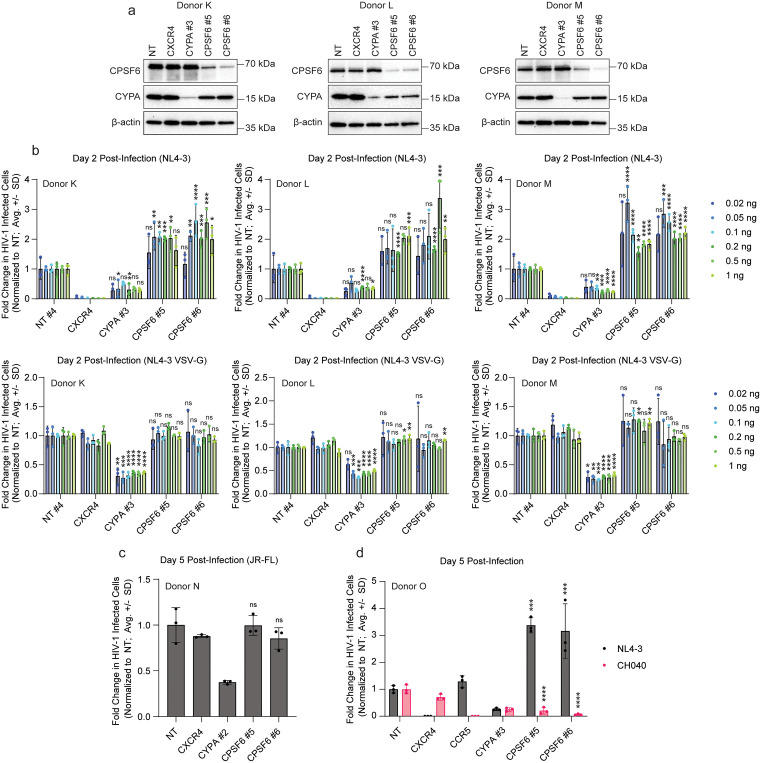
HIV-1 infection phenotypes in *CPSF6* knock-out cells vary by viral strain. a, Immunoblots shows knock-out of *CPSF6* and *CYPA* in primary CD4+ T cell protein lysates harvested at day 4 post-editing in 3 biological replicates (donors K-M). b, HIV-1 infectivity (% GFP positive cells normalized to NT control per condition) at day 2 post-challenge with HIV-1 NL4-3 nef:IRES:GFP (top) or VSV-G pseudotyped HIV-1 NL4-3 nef:IRES:GFP (bottom) in indicated knock-out primary CD4+ T cells from 3 biological replicates (donors K-M) as assessed by flow cytometry. Cells were treated infected with virus across a range of concentrations standardized by p24 content. Each bar represents the average of technical triplicates + /- SD with individual data points shown. Statistics were calculated relative to the NT control per condition by one-way ANOVA with Dunnet’s test for multiple comparisons; * = p ≤ 0.05, ** = p ≤ 0.01, *** = p ≤ 0.001, **** = p ≤ 0.0001. c, HIV-1 infectivity (% GFP positive cells normalized to NT control per condition) at day 5 post-challenge with HIV-1 JR-FL iGFP in indicated knock-out primary CD4+ T cells from 1 biological replicate (donor N) as assessed by flow cytometry. Each bar represents the average of technical triplicates + /- SD with individual data points shown. Statistics were calculated relative to the NT control by one-way ANOVA with Dunnet’s test for multiple comparisons; *** = p ≤ 0.001, **** = p ≤ 0.0001. d, HIV-1 infectivity (% GFP or FITC positive cells normalized to NT control per condition) at day 5 post-challenge with HIV-1 NL4-3 nef:IRES:GFP or CH040 in indicated knock-out primary CD4+ T cells from 1 biological replicate (donor O) as assessed by flow cytometry. Each bar represents the average of technical triplicates + /- SD with individual data points shown. Statistics were calculated relative to the NT control per condition by one-way ANOVA with Dunnet’s test for multiple comparisons; ns = not significant.

Given the increase in CXCR4 cell surface expression in the *CPSF6* knock-out cells, we next wanted to assess the impact of tropism on the observed infection phenotypes. To test this, we again repeated our knock-outs of *CPSF6* using two independent guides in 3 independent donors alongside NT, *CXCR4*-targeting, and *CYPA*-targeting controls (validated by immunoblot, Fig 3a). Cells were subsequently challenged in technical triplicate at 4 days post-electroporation with p24-normalized amounts of either replication-competent HIV-1 NL4-3 nef:IRES:GFP (as used before) or VSV-G pseudotyped, replication-incompetent HIV-1 NL4-3 dEnv nef:IRES:GFP. Percent infected (GFP+) cells were monitored by flow cytometry at 2 days post-infection and normalized to the NT control (Fig 3b). While the replication-competent virus again showed a 2- to 3-fold increase in replication in *CPSF6* knock-out cells, there was no significant difference in infection with the VSV-G pseudotyped virus (as previously reported [21]). This suggests that enhanced co-receptor expression is at least in part responsible for the increased permissivity.

To determine whether the HIV-1 infection phenotype in *CPSF6* knock-out cells is strain specific, we repeated our virus challenge experiments with the lab-adapted, CCR5-tropic HIV-1 JR-FL iGFP strain (Fig 3c) and the CCR5-tropic transmitted founder HIV-1 CH040 strain (Fig 3d). As before, we observed increased infection with our CXCR4-tropic NL4–3 strain at day 5 post-infection in the *CPSF6* knock-out cells (Fig 3d). However, we observed no significant impact of *CPSF6* knock-out on infection with the CCR5-tropic JR-FL strain. Surprisingly, we observed a strong decrease in infection with the CCR5-tropic transmitted founder strain upon *CPSF6* knock-out. These results indicate that the *CPSF6* knock-out phenotype in primary CD4+ T cells is strain-dependent and that the increased infection observed with the CXCR4-tropic HIV-1 NL4-3 strain is at least in part due to the increased cell surface expression of CXCR4.

### The interferon response is dampened in *CPSF6* knock-out primary CD4+ T cells

**Fig 4:**
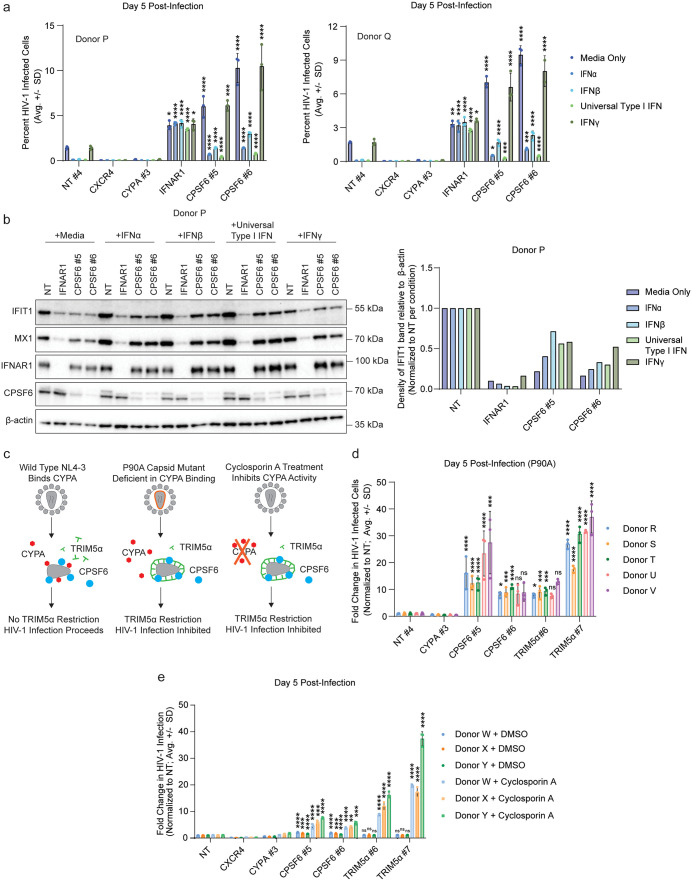
CPSF6 regulates the interferon pathway and expression of HIV-1 restriction factor TRIM5α in primary CD4+ T cells. a, HIV-1 infectivity (Raw % GFP positive cells normalized to NT control per condition) at day 5 post-challenge with HIV-1 NL4-3 nef:IRES:GFP in indicated knock-out primary CD4+ T cells from 2 biological replicates (donors P and Q) as assessed by flow cytometry. Cells were pre-treated with 100 U/mL IFNα, IFNβ, IFNγ, Universal Type I IFN, or media only control. Each bar represents the average of technical triplicates + /- SD with individual data points shown. Statistics were calculated relative to the NT control per condition by one-way ANOVA with Dunnet’s test for multiple comparisons; * = p ≤ 0.05, ** = p ≤ 0.01, *** = p ≤ 0.001, **** = p ≤ 0.0001. b, Immunoblot (left) shows expression of representative ISGs (IFIT1 and MX1) in knock-out primary CD4+ T cells treated with 10 U/mL IFNα, IFNβ, Universal Type I IFN, IFNγ, or media-only control for 16 hours in 1 representative biological replicate (donor P). Chart (right) shows IFIT1 band density divided by β-actin band density in adjacent blot. Density measurements are normalized to NT controls per condition. c, Diagram depicts methods of altering TRIM5α-mediated restriction in CD4+ T cells. Left: Wild-type HIV-1 NL4-3 capsid binds CYPA, which shields from TRIM5α restriction. Middle: The P90A capsid mutant HIV-1 derived from NL4-3 nef:IRES:GFP is deficient in CYPA binding, leading to TRIM5α-mediated restriction. Right: Cyclosporin A (CsA) inhibits CYPA activity, leading to TRIM5α-mediated restriction. d, HIV-1 infectivity (% GFP positive cells normalized to NT control per donor) at day 5 post-challenge with HIV-1 NL4-3 nef:IRES:GFP P90A capsid mutant in indicated knock-out primary CD4+ T cells from 5 biological replicates (donors R-V) as assessed by flow cytometry. Each bar represents the average of technical triplicates + /- SD with individual data points shown. Statistics were calculated relative to the NT control per condition by one-way ANOVA with Dunnet’s test for multiple comparisons; * = p ≤ 0.05, ** = p ≤ 0.01, *** = p ≤ 0.001, **** = p ≤ 0.0001. e, HIV-1 infectivity (% GFP positive cells normalized to NT control per condition) at day 5 post-challenge with HIV-1 NL4-3 nef:IRES:GFP in indicated knock-out primary CD4+ T cells from 3 biological replicates (donors W-Y) as assessed by flow cytometry. Cells were pre-treated with cyclosporin A or a DMSO control 4 hours prior to infection. Each bar represents the average of technical triplicates + /- SD with individual data points shown. Statistics were calculated relative to the NT control per condition by one-way ANOVA with Dunnet’s test for multiple comparisons; * = p ≤ 0.05, ** = p ≤ 0.01, *** = p ≤ 0.001, **** = p ≤ 0.0001.

Besides enhanced co-receptor expression, *CPSF6* knock-out cells also demonstrated broad transcriptional downregulation of the interferon signaling pathway. To test if this downregulation was functionally related to the observed increase in HIV-1 permissivity, primary CD4+ T cells from three independent donors were electroporated with 2 independent crRNPs targeting *CPSF6* alongside NT, *CXCR4*-targeting, and *CYPA*-targeting controls as well as a multiplexed pool of 5 crRNPs targeting the type I interferon receptor gene (*IFNAR1*). Cells were then pre-treated with media alone or 10 U/mL IFNα, IFNβ, Universal type I IFN, or IFNγ for 16 hours prior to challenge with replication-competent HIV-1 NL4-3 nef:IRES:GFP. At 5 days post-challenge, in the media only condition, *CXCR4* and *CYPA* knock-out decreased infection while *CPSF6* knock-out increased infection as expected (Figs 4a and S3a). Knock-out of *IFNAR1* resulted in a roughly two-fold increase in infection over the NT control compared to the 4- to 5-fold increase observed in the *CPSF6* knock-out cells. IFNα, IFNβ, and Universal type I IFN treatment decreased infection in the NT cells while IFNγ had no significant impact. The *IFNAR1* knock-out cells were not impacted by IFN treatment as expected. Notably, *CPSF6* knock-out cells showed a dampened response to IFNα, IFNβ, and Universal type I IFN treatment with a partial rescue of HIV-1 replication in these cells. Protein lysates collected after interferon pre-treatment, but prior to infection, showed decreased levels of representative ISGs IFIT1 and MX1 upon *CPSF6* knock-out, though not to the same extent as observed upon *IFNAR1* knock-out (Figs 3b and S3c).

To determine if the dampened IFN sensitivity in *CPSF6* knock-out cells depended on the cell entry pathway, we repeated the IFN treatment experiment using VSV-G pseudotyped, replication-incompetent HIV-1 NL4-3 dEnv nef:IRES:GFP alongside replication-competent HIV-1 NL4-3 nef:IRES:GFP and a wider range of doses of IFNα and IFNβ (1-1000 U/mL). Replication-competent HIV-1 NL4-3 showed enhanced infection in *CPSF6* knock-out cells compared to the NT control across all tested IFNα doses and the majority of IFNβ doses (S3b Fig). In contrast, VSV-G pseudotyped HIV-1 infection rates in *CPSF6* knock-out cells were similar to infection rates in NT control cells across all tested conditions (S3b Fig). This reaffirms our prior data that the increase in CXCR4 expression is a major driver of the observed increase in permissivity (Figs 2g and 3b), though it is also possible that this result stems from differential sensitivity of VSV-G pseudotyped viruses to inhibition by IFN. Taken together, these data suggest that *CPSF6* knock-out cells have a reduced responsiveness to IFN, which may in part explain the enhanced permissivity of *CPSF6* knock-out cells to HIV-1 infection.

Our RNA-Seq data also showed downregulation of a number of innate immune effectors and known HIV-1 restriction factors upon *CPSF6* knock-out, including TRIM5α, which binds to and restricts incoming cores. To protect itself from TRIM5α-mediated restriction, incoming cores bind CYPA, which masks the TRIM5α binding site [2,3]. Disruption of CYPA binding either by *CYPA* knock-out (as demonstrated previously [2,3] and shown above), mutation of the capsid (*i.e.*, through a capsid P90A mutation), or through treatment with the CYPA inhibitor cyclosporin A (CsA) decreases HIV-1 infection due to TRIM5α-mediated restriction (Fig 4c). To determine if *CPSF6* knock-out cells were functionally defective in TRIM5α, we electroporated primary CD4+ T cells from 5 independent donors with 2 independent crRNPs targeting *CYPA*, *CPSF6*, or *TRIM5α* alongside a NT control (immunoblot validation in S3d Fig). Cells were then challenged with HIV-1 NL4-3 nef:IRES:GFP containing a P90A capsid mutant in technical triplicate. Across all donors, *CYPA* knock-out had no significant effect on P90A virus infection, consistent with the inability of P90A capsids to recruit CYPA. Knock-out of *TRIM5α* led to a 10- to 30-fold increase in infection of the P90A virus, roughly correlating with knock-out efficiency as assessed by immunoblot (Figs 4d, raw infectivity data in S3e, immunoblot in S3d), consistent with prior reports [3]. Knock-out of *CPSF6* led to a decrease in TRIM5α steady-state levels across all donors (S3d Fig) and rescued P90A virus replication to similar levels as *TRIM5α* knock-out itself (Fig 4d).

To further confirm that *CPSF6* knock-out leads to restoration of infectivity under conditions that enhance TRIM5α-mediated restriction, we knocked out *CPSF6* and *TRIM5α* using 2 independent guides in 3 additional donors alongside NT, *CXCR4*-targeting, and *CYPA*-targeting controls (immunoblot validation in S3f Fig). Each population was challenged with HIV-1 NL4-3 nef:IRES:GFP in the presence and absence of CsA in technical triplicate. In the absence of CsA, *CYPA* knock-out decreased infection, *CPSF6* knock-out increased infection, and *TRIM5α* knock-out had no effect (Fig 4e). CsA strongly inhibited replication in the NT cells, though this was rescued in the *TRIM5α* knock-out cells with the degree of rescue correlating with knock-out efficiency (infection data in Figs 4e, knock-out efficiency in S3f). *CPSF6* knock-out partially rescued replication in the presence of CsA, consistent with the P90A capsid mutant data. These data suggest that the broad transcriptional reprogramming upon *CPSF6* knock-out leads to enhanced co-receptor expression, decreased interferon signaling, and reduced restriction factor expression, all of which partially contribute to enhanced HIV-1 replication in these cells.

### *CPSF6* knock-out in primary CD4+ T cells leads to changes in alternative polyadenylation

**Fig 5:**
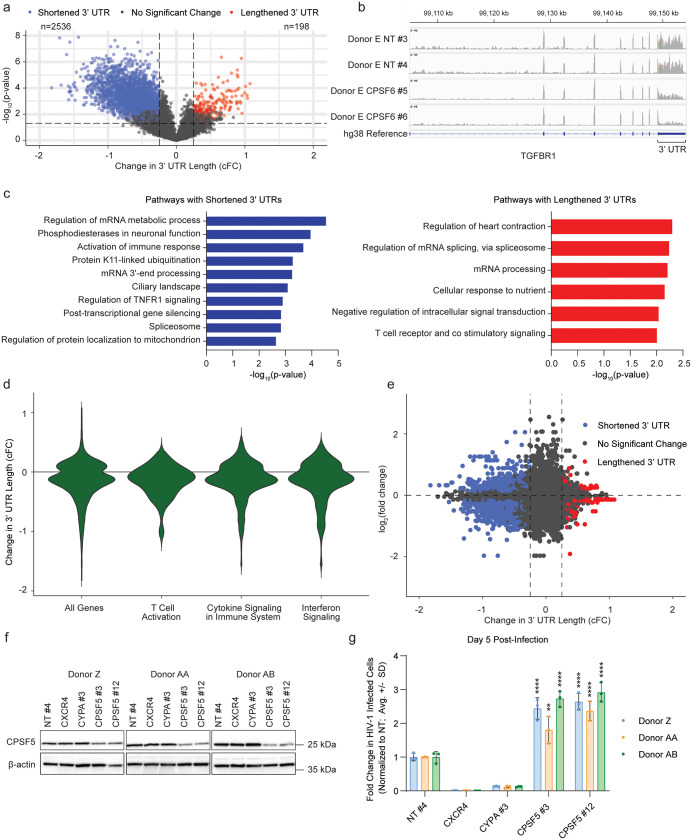
*CPSF6* knock-out leads to changes in alternative polyadenylation in primary CD4+ T cells a, Volcano plot shows REPAC analysis of changes in 3’ UTR lengths for *CPSF6* knock-out primary CD4 + T cells as compared to NT controls in 3 biological replicates (donors E-G, RNA-Seq data from Fig 2). 3’ UTRs are designated shortened (blue) for Compositional Fold Change (cFC) < -0.25 and -log_10_(adjusted p-value) < 0.05 and lengthened (red) for cFC  > 0.25 and -log_10_(adjusted p-value) < 0.05. Dots represent individual altered 3’ UTRs. b, Tracks show read coverage of TGFBR1 in RNA-Seq data from *CPSF6* knock-out and NT control cells from one representative biological replicate (donor E), visualized using Integrative Genomics Visualizer. 3’ UTRs show evidence of shortening in response to *CPSF6* knock-out. c, Charts show functional enrichment (Metascape) analysis of top pathways enriched in genes with shortened 3’ UTRs (left, blue) and lengthened 3’ UTRs (right, red) in response to *CSPF6* knock-out in primary CD4+ T cells as assessed by REPAC analysis in 3 biological replicates (donors E-G). d, Violin plot shows comparison of cFC values from REPAC analysis in all tested genes as compared to genes in the indicated pathways (GO:0042110:T Cell activation, R-HSA-1280215: Cytokine Signaling in Immune system, R-HSA-913531: Interferon Signaling) in 3 biological replicates (donors E-G). e, Scatterplot shows cFC of all tested APA sites in REPAC analysis of *CPSF6* knock-out primary CD4+ T cells in as compared to NT controls plotted against differential expression data as shown in Fig 2 in data aggregated from 3 biological replicates (donors E-G). 3’ UTRs are designated shortened (blue) for cFC < -0.25 and -log_10_(adjusted p-value) < 0.05 and lengthened (red) for cFC > 0.25 and -log_10_(adjusted p-value) < 0.05. Dots represent individual altered 3’ UTRs. f, Immunoblot shows knock-out of *CPSF5* in protein lysates harvested at day 4 post-editing in 3 biological replicates (donor Z-AB). g, HIV-1 infectivity (% GFP positive cells normalized to NT control per donor) at day 5 post-challenge with HIV-1 NL4-3 nef:IRES:GFP in indicated knock-out primary CD4+ T cells from 3 biological replicates (donors Z-AB) as assessed by flow cytometry. Each bar represents the average of technical triplicates + /- SD with individual data points shown. Statistics were calculated relative to the NT control per condition by one-way ANOVA with Dunnet’s test for multiple comparisons; * = p ≤ 0.05, ** = p ≤ 0.01, *** = p ≤ 0.001, **** = p ≤ 0.0001.

Given that CPSF6 regulates polyadenylation site selection through its role in the CFIm complex, we hypothesized that the broad transcriptional changes in primary CD4+ T cells upon *CPSF6* knock-out could be driven by changes in APA. To test this hypothesis, we re-analyzed our RNA-Seq data from uninfected *CPSF6* knock-out primary CD4+ T cells using Regression of PolyAdenylation Compositions (REPAC) analysis [36]. Briefly, this algorithm compares RNA-Seq reads on either side of known, annotated poly(A) sites and calculates the compositional Fold Change (cFC) for each gene as a measure of 3’ UTR length changes between an experimental (*CPSF6* knock-out) and control (NT) condition. Shortening of 3’ UTRs is indicated by a cFC value < -0.25, and lengthening by a value > 0.25. REPAC analysis revealed that *CPSF6* knock-out primary CD4+ T cells exhibited an overall bias towards shortening of 3’ UTR lengths (n = 2,536 shortened, n = 198 lengthened 3’ UTRs) as compared to NT controls, consistent with what has been observed in other cell types [16] (Fig 5a). These changes in 3’ UTR lengths can be readily observed in the transcript read coverage tracks, as shown at the transforming growth factor-beta receptor type 1 (TGFBR1) locus, for example (Fig 5b). Functional enrichment analysis of genes with significantly shortened 3’ UTRs identified broad changes in APA at genes associated with immune response signaling, suggesting that shortening of these transcripts may be associated with their downregulation (Fig 5c). To validate this result, we plotted the cFC values for all genes previously identified in the functional enrichment analysis associated with the term ‘Interferon Signaling’ (Fig 2f) and found these genes showed clear evidence of 3’ UTR shortening (Fig 5d).

A decrease in 3’ UTR length was not universally associated with decreased transcript abundance. Indeed, similar decreases in cFCs were observed for transcripts associated with the ‘Cytokine Signaling in Innate Immune Signaling’ and ‘T Cell Activation’ pathways, both of which were enriched pathways among the upregulated genes (Figs 5c and 2f). To assess the relationship between changes in 3’ UTR length and transcript abundance more globally, we plotted cFC values from all analyzed poly(A) sites against the associated log_2_(fold change) values from the differential gene expression analysis in Fig 2. While there was a bias towards genes with shortened 3’ UTRs exhibiting downregulation, changes in 3’ UTR length were associated with variable changes in transcript abundance, which suggests multiple modes of gene-dependent regulation (Fig 5e). Additionally, several genes with significant changes in abundance did not exhibit a significant change in 3’ UTR length, suggesting there are additional and/or secondary effects of *CPSF6* knock-out on the transcriptome by this timepoint (Fig 5e). *CXCR4* and *CD25*, for example, were upregulated and had increased cell surface expression (Fig 2g), but only *CD25* showed a significant decrease in 3’ UTR length (S4a Fig).

Several different algorithms have been developed to assess poly(A) site usage, all of which use slightly different approaches. The REPAC algorithm uses only well-annotated poly(A) sites, but as a result does not give information on every locus. Therefore, to validate these results across the transcriptome, we leveraged an alternative method of APA analysis, Dynamic analysis of Alternative PolyAdenylation (DaPars) [37], that infers poly(A) site usage and calculates changes in the distal polyadenylation usage index (ΔPDUI) as a proxy for changes in 3’ UTR length for each gene (S4b Fig). As with the REPAC analysis, ΔPDUI analysis showed that *CPSF6* knock-out led to global 3’ UTR shortening, with genes involved in the interferon pathway, cytokine signaling, and cellular activation showing preferential 3’ UTR shortening (S4c Fig).

These data suggest that changes in APA are induced by *CPSF6* knock-out and are at least partially responsible for the broad transcriptional reprogramming and increased permissivity to HIV-1 observed in these cells. If this is true, we would hypothesize that knock-out of the other CFIm complex member, CPSF5, would result in a similar phenotype, as *CPSF5* knock-out has also previously been reported to trigger changes in APA [38]. To test this hypothesis, we knocked out *CPSF5* in primary CD4+ T cells using 2 independent guide RNA in 3 independent donors and conducted HIV-1 spreading infection assays as before alongside NT, *CXCR4* knock-out, and *CYPA* knock-out controls. *CPSF5* knock-out had minimal impact on cell viability (S4d Fig). While *CPSF5* knock-out efficiency wasn’t as high as that achieved for *CPSF6* as monitored by immunoblot (Fig 5f), it still resulted in a significant increase in HIV-1 infection (Fig 5g). Taken together, these data suggest that the induction of APA through CFIm complex disruption results in broad transcriptional reprogramming and enhanced CD4+ T cell permissivity to HIV-1 infection.

### HIV-1 capsid binding to CPSF6 triggers transcriptional rewiring

Previously described roles of CPSF6 in HIV-1 infection, including its roles in nuclear import and integration site selection, are dependent on CPSF6 binding to HIV-1 cores. Several studies have shown that this interaction can also lead to relocalization of CPSF6 to nuclear speckles, with the extent of relocalization dependent on the cell type and timepoint [39–42]. This is dependent on the interaction with the core as infection with the N74D capsid mutant, which does not bind CPSF6, does not lead to CPSF6 relocalization to nuclear speckles [42]. It is unclear, however, if the interaction of CPSF6 with HIV-1 capsid and its subsequent relocalization to nuclear speckles also disrupts CFIm function and triggers changes in APA.

To test this, we first used the HT1080 cell line model as recruitment of CPSF6 to nuclear speckles had previously been reported following HIV-1 infection in these cells [40]. To more readily track changes in CPSF6 localization, we engineered an endogenous monomeric NeonGreen (mNGreen) C-terminal tag into the CPSF6 locus in HT1080 cells via CRISPR-Cas9 knock-in (HT1080-CPSF6-mNGreen). We challenged these HT1080-CPSF6-mNGreen cells with VSV-G pseudotyped HIV-1 NL4-3 dEnv encoding either a wild-type (WT) capsid or a capsid mutant that is deficient in CPSF6 binding (N74D) at a multiplicity of infection (MOI) of 10 in technical triplicate. At 6 hours post-challenge, we performed immunofluorescent staining for a marker of nuclear speckles, SC35, in tandem with harvesting RNA and protein for analysis by bulk RNA-Seq and immunoblotting, respectively. Widefield deconvolution fluorescent microscopy imaging confirmed that infection with WT HIV-1 led to the formation of CPSF6 puncta in cell nuclei, which were not observed in the mock or N74D infected cells (S5a Fig). The CPSF6 puncta in the WT HIV-1 infected cells were largely colocalized with SC35-containing nuclear speckles, consistent with prior reports (S5a and quantification in S5b Fig). Immunoblotting of protein lysates harvested from the infected cells demonstrated that CPSF6 protein levels were unchanged between the mock, WT, and N74D infection conditions (S5c Fig).

**Fig 6:**
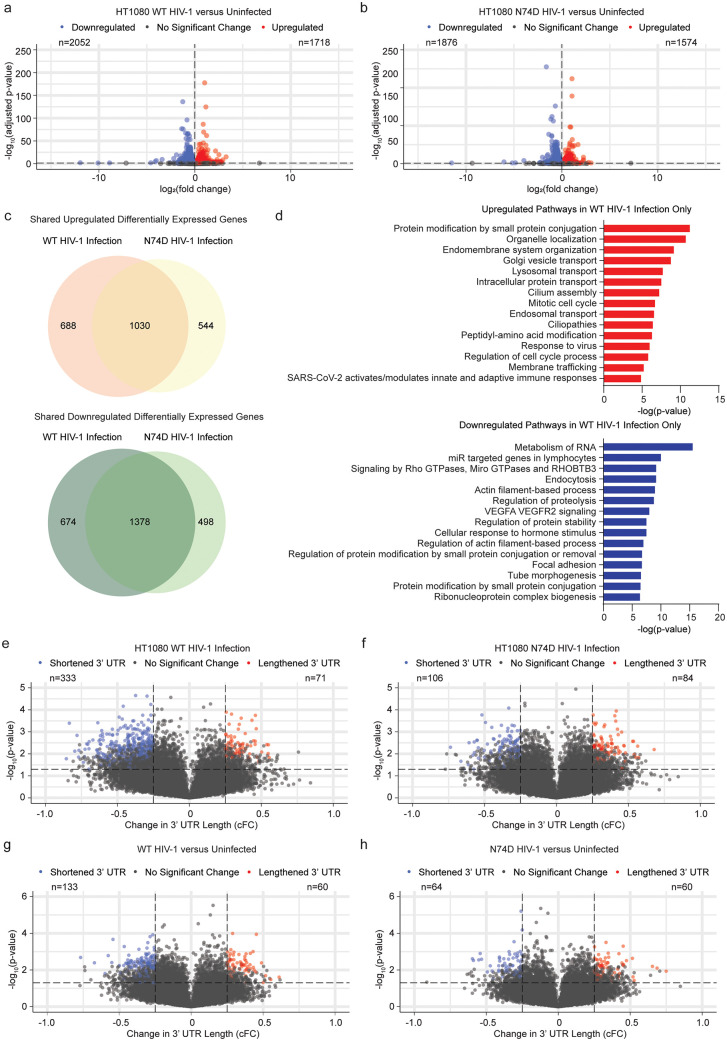
HIV-1 infection induces APA in HT1080 cells a, Volcano plot shows differentially expressed genes in RNA-Seq data from HT1080-CPSF6-mNGreen cells infected with VSV-G pseudotyped WT HIV-1-NL4-3 at an MOI of 10 at 6 hours post-infection as compared to mock-infected controls in 3 technical replicates. Dots show significantly downregulated (blue) and upregulated (red) genes, dotted line shows threshold of 0.05 for adjusted p-value. b, Volcano plot shows differentially expressed genes in RNA-Seq data from HT1080-CPSF6-mNGreen cells infected with VSV-G pseudotyped N74D capsid mutant HIV-1-NL4-3 at an MOI of 10 at 6 hours post-infection as compared to mock-infected controls in 3 technical replicates. Dots show significantly downregulated (blue) and upregulated (red) genes, dotted line shows threshold of 0.05 for adjusted p-value. c, Venn diagrams shows overlap of upregulated (top) and downregulated (bottom) differentially expressed genes between WT infected and N74D infected HT1080-CPSF6-mNGreen cells in 3 technical replicates. d, Charts show functional enrichment (Metascape) analysis of top 15 upregulated (top, red) and downregulated (bottom, blue) pathways in differentially expressed genes from WT infected HT1080-CPSF6-mNGreen cells that are not differentially expressed in N74D infected HT1080-CPSF6-mNGreen cells at 6 hours post-infection in 3 technical replicates. e, Volcano plot shows REPAC analysis of changes in 3’ UTR lengths for WT VSV-G-pseudotyped HIV-1-NL4-3-infected versus mock-infected HT1080-CPSF6-mNGreen cells from 3 technical replicates. Cells were infected at an MOI of 10 and RNA was extracted at 6 hours post-infection. 3’ UTRs are designated shortened (blue) for Compositional Fold Change (cFC) < -0.25 and -log10(adjusted p-value) < 0.05 and lengthened (red) for cFC > 0.25 and -log10(adjusted p-value) < 0.05. Dots represent individual altered 3’ UTRs. f, Volcano plot shows REPAC analysis of changes in 3’ UTR lengths for N74D VSV-G-pseudotyped HIV-1-NL4-3-infected versus mock-infected HT1080-CPSF6-mNGreen cells from 3 technical replicates. Cells were infected at an MOI of 10 and RNA was extracted 6 hours post-infection. 3’ UTRs are designated shortened (blue) for cFC < -0.25 and -log10(adjusted p-value) < 0.05 and lengthened (red) for cFC > 0.25 and -log10(adjusted p-value) < 0.05. Dots represent individual altered 3’ UTRs. g, Volcano plot shows REPAC analysis of changes in 3’ UTR lengths for WT HIV-1 NL4-3 nef:IRES:GFP infected versus uninfected primary CD4+ T cells from 3 biological replicates (donors AC-AE). 3’ UTRs are designated shortened (blue) for cFC < -0.25 and -log_10_(adjusted p-value) < 0.05 and lengthened (red) for cFC > 0.25 and -log_10_(adjusted p-value) < 0.05. Dots represent individual altered 3’ UTRs. h, Volcano plot shows REPAC analysis of changes in 3’ UTR lengths for N74D capsid mutant HIV-1 NL4-3 nef:IRES:GFP infected versus uninfected primary CD4+ T cells from 3 biological replicates (donors AC-AE). 3’ UTRs are designated shortened (blue) forcFC < -0.25 and -log_10_(adjusted p-value) < 0.05 and lengthened (red) for cFC > 0.25 and -log_10_(adjusted p-value) < 0.05. Dots represent individual altered 3’ UTRs.

We next sought to profile the transcriptional changes in our WT or N74D infected HT1080 cells relative to our mock infected controls at 6 hours post-challenge. Challenge with WT HIV-1 resulted in 3,770 differentially expressed genes (Fig 6a), whereas challenge with the N74D virus resulted in slightly fewer changes with 3,450 differentially expressed genes (Fig 6b). While a majority of differentially expressed genes are shared in both infection conditions, 1,362 differentially expressed genes were found only in the WT, but not N74D, infected condition (Fig 6c). Functional enrichment analysis of this set of genes revealed upregulation of several pathways involved in organelle organization and membrane trafficking and downregulation of several pathways involved in RNA metabolism and actin filament regulation (Fig 6d).

To assess the impact of infection on APA, we next performed REPAC analysis on the RNA-Seq data from the HT1080 cells. The HT1080 cells challenged with WT HIV-1 demonstrated a bias towards an overall shortening of 3’ UTR lengths as compared to mock-infected controls (333 shortened, 71 lengthened; Fig 6e). On the other hand, HT1080 cells challenged with the N74D capsid mutant HIV-1 virus did not show a directional bias in 3’ UTR lengths as compared to mock-infected controls (108 shortened, 84 lengthened; Fig 6f). These results suggest that binding of CPSF6 to HIV-1 capsid cores and/or its subsequent relocalization is sufficient to partially perturb CFIm function and result in changes to APA.

To determine whether HIV-1 infection induces changes in APA and transcriptional reprogramming through recruitment of CPSF6 to capsid cores in a more physiologically relevant cell type, we next challenged primary CD4+ T cells from 3 independent donors with HIV-1 NL4-3 nef:IRES:GFP encoding either a WT or N74D mutant capsid. The N74D capsid mutant is known to have a severe replication defect in primary CD4+ T cells [22,23]. To account for this, we performed live cell sorting on infected (GFP+) cells at day 2 post-challenge to obtain pure populations of infected cells, thus mitigating differences in infection rates between the two viruses. RNA was then extracted from the sorted cell populations alongside uninfected controls and subjected to RNA-Seq (S6a Fig). In parallel, we generated *CPSF6* knock-out and NT control primary CD4+ T cells and extracted RNA at 2 days post-electroporation to directly compare the transcriptional profiles between the infected cells at day 2 post-infection and *CPSF6* knock-out cells at day 2 post-editing (immunoblot validation of knock-out efficiency in S6b Fig).

As reported in Fig 2, *CPSF6* knock-out resulted in broad transcriptional rewiring of primary CD4+ T cells with 3,579 differentially expressed genes (S6c Fig), including upregulation of genes involved in cytokine signaling and downregulation of those involved in the innate immune response (S6d Fig). As observed in the HT1080 cells, infection with WT virus resulted in broad transcriptional changes with 4,537 differentially expressed genes (S6e Fig) while infection with the N74D virus resulted in slightly fewer changes with 3,787 differentially expressed genes (S6f Fig). Comparing between conditions, the WT infected and *CPSF6* knock-out cells shared 757 misregulated genes, while the N74D infected and *CPSF6* knock-out cells shared 640 misregulated genes (S6g Fig). While the two infected conditions were more similar, the cells with WT infected virus had 1,650 differentially expressed genes not shared by the cells infected with N74D mutant virus. Functional enrichment analysis of these genes showed upregulation of RNA metabolism and cell cycle pathways and downregulation of membrane trafficking and organelle localization pathways, contrary to what was observed in the infected HT1080 cells (S6h Fig).

To assess if these changes were associated with changes in APA, we next performed REPAC analysis on the RNA-Seq data from the *CPSF6* knock-out and infected primary cells. The *CPSF6* knock-out cells, as before, showed a strong bias towards overall shortening of 3’ UTR lengths with 928 shortened and only 50 lengthened 3’ UTRs (S6c Fig). The primary T cells infected with WT virus showed a similar, but much smaller, bias towards overall 3’ UTR shortening as compared to uninfected controls (133 shortened, 60 lengthened; Fig 6g, individual gene names provided in S6i Fig). On the contrary, cells infected with the N74D virus did not show a directional bias in 3’ UTR lengths as compared to uninfected controls (64 shortened, 60 lengthened; Fig 6h). These data were independently confirmed using the ΔPDUI algorithm, which likewise confirmed a bias towards 3’ UTR shortening in the cells infected with the WT virus (S6j Fig), but not the N74D virus (S6k Fig). Similar to the *CPSF6* knock-out data, a majority of gene expression changes cannot be directly linked to a change in 3’ UTR length at this timepoint, though this doesn’t rule out secondary or indirect effects of APA.

If transcriptional rewiring triggered by CPSF6 recruitment to capsid cores enhances CD4 + T cell permissivity to infection, we would expect that mutations that prevent CPSF6 binding would decrease infectivity, as has been reported with the N74D capsid mutant virus [22,23], and that knock-out of *CPSF6* would help rescue infection of those viruses. To test this, we electroporated primary CD4+ T cells from 5 independent donors with 2 independent crRNPs targeting *CPSF6* and *TRIM5α* alongside a NT and *CYPA*-targeting control (immunoblot validation in S3c Fig). Cells were then challenged with HIV-1 NL4-3 nef:IRES:GFP containing the N74D capsid mutant in technical triplicate. At day 5 post-challenge, *CPSF6* knock-out cells showed a 4- to 6-fold increase in infection relative to the NT controls, indicating that knock-out of *CPSF6* enhances infectivity of the N74D capsid mutant (S6l Fig). Previous reports have shown the N74D capsid mutant also results in reduced binding of CYPA [21], and that knock-out of *TRIM5α* was sufficient to rescue infection, which we also observe here (S6l Fig). The pleiotropic nature of the N74D capsid mutant, the previously demonstrated impact of CPSF6 on TRIM5α levels (Fig 4), and the large number of transcriptional changes induced by *CPSF6* knock-out complicates interpretation of this experiment. It may be that the downregulation of TRIM5α is primarily responsible for the observed rescue of N74D replication, though more would need to be done to follow up on this phenotype.

Taken together, these data are consistent with a model in which CPSF6 recruitment and relocalization by incoming capsid cores can trigger transcriptional reprogramming of infected cells through changes in APA to enhance permissivity to infection. The strain and cell type specificity of the observed infectivity changes upon *CPSF6* knock-out suggests a complex interplay between the viral and host determinants which should be carefully considered when interpreting prior and future experimental data on this host-pathogen interaction.

## Discussion

The known functions of CPSF6 in nuclear import and integration site selection are not sufficient to explain the enhanced permissivity to HIV-1 infection observed upon *CPSF6* knock-out in primary CD4+ T cells. While the magnitude of this effect varies slightly by donor, we observed increased HIV-1 NL4-3 infection upon *CPSF6* knock-out in all 41 independent donors used in this study. Smaller increases in infection upon *CPSF6* knock-down have also been observed in other cell types, such as HEK293T and HeLa cells [16,20]. We propose a model wherein CPSF6 perturbation results in broad transcriptional reprogramming and enhanced permissivity to infection, which we demonstrate is mediated in part by enhanced receptor expression and a dampened interferon response. Given this multifaceted mechanism-of-action, we would expect this phenotype to be highly dependent on experimental set up, with the cell type, viral tropism, and baseline immune activation all influencing the magnitude of the effect. This is in line with prior reports showing that basal IFN signaling and HIV-1 sensing in primary CD4+ T cells varies on a donor-to-donor basis. It remains to be seen if there are additional cell type dependencies in the subsets of genes regulated by CPSF6 and what other host factors may dictate this selectivity.

Previous studies of HIV-1 infection in *CPSF6* knock-out or knock-down cells primarily relied on immortalized cell line models that do not fully recapitulate key features of HIV-1 infection [43]. Our approach for CRISPR-Cas9 knock-out of *CPSF6* in primary CD4+ T cells involves the electroporation of crRNPs into cells to generate polyclonal knock-out cell pools, an approach that carries benefits and drawbacks. Given that repair of the CRISPR-induced double-strand break is stochastic, this method can result in alleles with insertions, deletions, or in-frame repairs that retain protein expression. Thus, the polyclonal pool of cells is actually a mixture of homozygous knock-out cells expressing no CPSF6, homozygous “wild-type” cells expressing CPSF6, and heterozygous cells expressing only some CPSF6. Since it is not possible to isolate monoclonal cell populations from primary cells, this residual amount remains a limitation of the approach, and we monitor protein levels in every experiment to ensure efficient knock-out prior to phenotypic characterization. We achieve nearly complete ablation of CPSF6 protein expression in a majority of our donors by the time spreading infection is initiated, particularly with CPSF6 guide #6. Unfortunately, it is generally not possible to obtain monoclonal *CPSF6* knock-out immortalized T cell lines, such as Jurkats, despite extensive efforts by multiple groups [13,44]. This is likely due to CPSF6 being required for cell survival in these cell lines, which is notably different than the primary T cells which show minimal viability effects from CPSF6 depletion. Thus, while residual CPSF6 protein expression is observed in the polyclonal pool of primary CD4+ T cells, we believe that a majority of cells are homozygous knock-outs and that these cell populations represent a highly physiologically relevant model for understanding CPSF6 function.

We also observed notable strain-specific differences in the phenotype with CXCR4-tropic, CCR5-tropic, and transmitted founder strains having different infection phenotypes in *CPSF6* knock-out cells. Neither the lab-adapted CCR5-tropic viruses nor the VSV-G pseudotyped virus showed a significant increase in infection, suggesting that that increased CXCR4 expression is the major driver of enhanced CXCR4-tropic virus permissivity in these cells. While decreased interferon sensitivity and restriction factor expression are functionally relevant (as shown by our interferon and TRIM5α experiments), these changes alone are likely insufficient to drive changes in wild-type virus replication under these conditions. The block to replication for the transmitted founder virus in *CPSF6* knock-out cells, however, is less clear. We confirmed this is not due to changes in CCR5 cell surface expression, though we have yet to map the stage of the lifecycle that is blocked. If CPSF6 is required for nuclear import or trafficking of this transmitted founder strain, this effect would be dominant to any observed increase in permissivity. However, this is clearly not the case for the lab-adapted strains, suggesting that there are additional viral determinants of the phenotype to be uncovered in future work.

One of the well-known functions of CPSF6 is its role in polyadenylation site selection as part of the CFIm complex with CPSF5. CFIm complex disruption through knock-out of *CPSF5* also led to enhanced permissivity to HIV-1 in our model, suggesting that changes in APA are directly responsible for the infection phenotype. Prior studies have demonstrated that disruption of this complex results in changes in APA and an overall shortening of 3’UTR lengths [28,29]. Consistent with this, we observe a strong bias towards 3’UTR shortening in primary CD4 + T cells upon *CPSF6* knock-out using 2 independent algorithms. While the effect of 3’UTR length on transcript abundance was variable, genes with shortened 3’UTRs were generally downregulated. Genes in the interferon signaling pathway, for example, had preferentially shortened 3’ UTRs and were generally downregulated. This is in line with previous studies that have reported a role for APA in the regulation of the innate immune response to infection with other viruses, including Sendai Virus, Influenza A Virus, and Vesicular Stomatitis Virus [32,45,46]. This is not universal, as other groups have reported that depletion of CPSF6 leads to upregulation of antiviral immune response in some cell lines [46], again pointing to important differences in cell type and experimental technique that may govern CPSF6-mediated phenotypes in response to viral infection.

Exactly how changes in APA lead to changes in steady-state expression of transcripts in shared pathways remains to be determined. One possibility is that changes in 3’ UTR length alter mRNA steady-state levels by removing binding sites for unspecified microRNAs and/or RNA binding proteins. That being said, the number of differentially expressed genes was substantially larger than the number of genes with altered 3’ UTR lengths. Some of the discrepancy between changes in 3’ UTR length and changes in transcript abundance may be due to limitations of the algorithms used for APA analysis. For example, REPAC analysis relies on previously annotated sites, and some genes still lack this annotation, preventing the algorithm from assessing shortening or lengthening for those transcripts. There may also be alternate, indirect means by which *CPSF6* knock-out may lead to transcriptional reprogramming, such as changes in 3’ UTR length leading to altered expression of a transcription factor, which in turn impacts the expression of other genes. Dedicated 3’-end sequencing approaches to more accurately profile changes in APA will be needed in the future to better assess this association.

Incoming HIV-1 capsid cores are known to bind CPSF6 at a conserved interface with defined roles in nuclear entry and integration site selection [47]. Furthermore, in some cell types, HIV-1 infection has been shown to induce relocalization of nuclear CPSF6 to sites of pre-mRNA processing known as nuclear speckles [39–42]. We reasoned that if CPSF6 binding to the capsid and/or relocalization was sufficient to induce changes in APA, it could represent a novel mechanism by which the virus reprograms its target cells to optimize its replication. Indeed, we found that in HT1080 cells, which we and others have shown to exhibit relocalization of CPSF6 to nuclear speckles following HIV-1 infection [40], there is a bias towards 3’ UTR shortening for wild-type infected cells, but not N74D infected cells, at 6 hours post-infection. We also observed a bias towards 3 ’UTR shortening in the wild-type infected primary CD4+ T cells, but not the N74D infected cells. That being said, the number of genes with significantly altered 3’ UTR lengths was much smaller in infected primary CD4+ T cells compared to *CPSF6* knock-out cells or HIV-1 infected HT1080 cells. Studies of CPSF6 puncta formation in response to HIV-1 infection have reported that cell types differ in initial localization of nuclear CPSF6 prior to infection, with cell types such as HT1080 cells exhibiting diffuse nuclear staining for CPSF6 in uninfected cells [39,40]. In contrast, a recent study observed colocalization of CPSF6 with nuclear speckles in primary CD4+ T cells even in the absence of HIV-1 infection, indicating that CPSF6 may undergo less dramatic changes in localization following infection of this cell type [48]. This could explain why we observed fewer changes in 3’ UTR lengths following wild-type HIV-1 infection in primary CD4 + T cells as compared to HT1080 cells and may point to potential cell-type differences in CPSF6 function. CPSF6 relocalization may also differ based on the cellular perturbation that is leading to changes in alternative polyadenylation. Notably, other groups have reported that CPSF6 relocalization to puncta within the nucleus is associated with preferential 3’ UTR lengthening in several cancer cell lines, pointing to other as-of-yet unexplored cell type differences that may influence CPSF6-mediated transcriptional reprogramming [49].

Alternately, besides cell type specific differences, it is also possible that viral dose and/or timing could impact the magnitude of the changes in APA observed in response to HIV-1 infection, with higher MOI challenges leading to larger effects, especially earlier in the lifecycle. Our studies do not fully address the time scale at which CPSF6-mediated transcriptional reprogramming occurs following HIV-1 infection. In our experiments, *CPSF6* knock-out leads to transcriptional changes that occur prior to virus infection, so the timescale of these transcriptional changes cannot necessarily be compared directly to the transcriptional changes that occur as a result of CPSF6 relocalization during the process of HIV-1 replication. For example, enhanced permissivity due to upregulation of CXCR4 would not be recapitulated by capsid-mediated alteration of CPSF6 function, since this interaction occurs after entry. However, other transcriptional changes we observe, such as diminished innate immune signaling and enhanced T cell activation, are more consistent with our model, but more mechanistic study of this is needed.

The changes in APA observed downstream of HIV-1 infection are not observed when we challenge cells with the capsid N74D mutant. While the N74D capsid mutant virus serves as a useful tool to study CPSF6 function, it is known to have pleiotropic impacts on the binding of other host factors, most notably CYPA. Due to the CYPA binding deficiency, the N74D capsid mutant is highly susceptible to TRIM5α restriction and replicates poorly in primary cell types [21]. Thus, it is possible that there are additional host factors that contribute to the observed phenotypes with this mutant that are not accounted for in this study. In particular, it would be of interest to compare these results with the capsid A77V mutant, which has likewise been shown to reduce CPSF6 binding to the core, but otherwise generally retains viral infectivity in diverse cell line models and primary cell types.

CPSF6 binding to incoming capsid cores has been previously suggested to shield the incoming virus from innate immune sensing in myeloid cells [19]. While our models differ, these data are highly complementary to our observations, with the critical difference being that knock-down of *CPSF6* in primary macrophages was observed to inhibit HIV-1 replication. This is consistent with data in several other cell lines wherein CPSF6 depletion results in decreased viral replication associated with defects in nuclear import [10]. Our data suggests that CPSF6 is not required for nuclear import in primary CD4+ T cells, though it remains to be seen if this is due to cell cycling or the role of some other redundant factor. In cell types where CPSF6 is required for nuclear import, we would expect this phenotype to be dominant, but this is not inconsistent with an additional role for CPSF6 recruitment in transcriptional reprogramming. This study highlights the magnitude of transcriptional changes that occur in *CPSF6* knock-out cells, and these transcriptional changes should be considered when interpreting HIV-1 infection phenotypes in *CPSF6* knock-out cells across other cell types.

Taken together, our data suggest a model in which CPSF6 recruitment by incoming capsid cores triggers transcriptional reprogramming of infected cells through APA to enhance permissivity to infection. This reprogramming may reflect a means by which HIV-1 achieves a targeted host shutoff to dampen innate immune sensing and promote viral replication. Future studies will focus on understanding the viral and host determinants that govern the multifaceted role of CPSF6 in HIV-1 infection.

## Methods

### Cell Lines

Human embryonic kidney (HEK) 293T cells were obtained from ATCC (#CRL-3216) and cultured in 1x Dulbecco’s Modified Eagle Medium (DMEM) (Corning, #10–013-CV) supplemented with 10% heat inactivated fetal bovine serum (Gibco, #16140–071) and 1% penicillin/streptomycin (Cytiva, #SV30010). 293AAV (Cell Biolabs, Cat# AAV-100) and HT1080 (ATCC, #CVCL_0317) cell lines were maintained in 1x DMEM (ThermoFisher, 11995065), supplemented with 10% fetal calf serum (Sigma, #F092) and gentamicin (Gibco, #15750060). Cells were maintained in a humidified cell culture incubator at 37°C with 5% CO_2_.

### Plasmid Constructs

An HIV-1 NL4-3 molecular clone with GFP cloned behind an IRES cassette following the viral *nef* gene (nef:IRES:GFP) was used as the base vector for all viral plasmids (BEI Resources, #11349). N74D and P90A capsid mutants were generated from the original plasmid using site-directed mutagenesis (SDM). Briefly, a portion of the original NL4-3 nef:IRES:GFP capsid region between the SfoI and SpeI restriction sites was amplified and cloned into a pJet1.2/blunt vector (Thermo Scientific, #K1231). The pJet1.2 plasmid with the capsid insert was used for SDM to make the desired point mutations with the following primers:[Table ppat.1013745.t001]

**Table ppat.1013745.t001:** 

Description	Sequence
HIV-1 Capsid SDM N74D Forward	5’-GTTAAAAGAGACCATCGATGAGGAAGCTGCAG-3’
HIV-1 Capsid SDM N74D Reverse	5’-CTGCAGCTTCCTCATCGATGGTCTCTTTTAAC-3’
HIV-1 Capsid SDM P90A Forward	5’-TCCAGTGCATGCAGGGGCTATTGCACCAGGC-3’
HIV-1 Capsid SDM P90A Reverse	5’-GCCTGGTGCAATAGCCCCTGCATGCACTGGA-3’

The PCR reactions were transformed into chemically competent *E. coli* (Takara Bio, #636763), and colonies were screened for the desired mutation by Sanger sequencing using the following primer sequences: 5’-CGACTCACTATAGGGAGAGCGGC-3’, 5’-AAGAACATCGATTTTCCATGGCAG-3’. Capsid region inserts bearing the desired mutation were isolated from the pJet1.2 vector and re-inserted into the original NL4-3 nef:IRES:GFP vector by Gibson assembly. Final plasmid sequences were confirmed via Sanger sequencing with the following primers: 5’-AGCGTCGGTATTAAGCGGGG-3’, 5’-ATTCCCTGGCCTTCCCTTGT-3’.

### Primary CD4+ T Cell Isolation and Culture

Primary CD4+ T cells were isolated from human peripheral blood leukopaks sourced from de-identified healthy donors (STEMCELL Technologies, #200–0092). Leukopak blood products were diluted to 150 mL in Dulbecco’s Phosphate-Buffered Saline (DPBS) (Corning, #21–031-CV) with 2mM EDTA (Corning, #46–034-Cl) per donor. PBMCs were isolated through Ficoll-Paque centrifugation (Cytiva, #17-5442-03) followed by washing with DPBS with 2mM EDTA. PBMCs were resuspended in MACS buffer (DPBS with 0.5% bovine serum albumin (BSA) (Fisher Scientific, #BP9706100), and 2mM EDTA), and CD4+ T cells were then isolated from PBMCs using an EasySep Human CD4+ T Cell Isolation Kit (STEMCELL Technologies, #17952) per the manufacturer protocol. Cells were resuspended at 2.5x10^6^ cells/mL in complete Roswell Park Memorial Institute (RPMI) medium consisting of RPMI 1640 (Corning, #10–040-CV) supplemented with 10% heat inactivated fetal bovine serum (Gibco, #16140–071), 12.5 mL HEPES (Cytiva, #SH30237.01), 5 mL sodium pyruvate (Corning, #25–000-CI), and 1% penicillin/streptomycin (Cytiva, #SV30010). Media was supplemented with interleukin-2 (IL-2) at 20 IU/mL immediately before use (Miltenyi, #130097744). Cells were activated on α-CD3-coated plates (Tonbo Biosciences, UCHT1) incubated at 4°C overnight before use, with soluble α-CD28 added at 5 mg/mL to media before plating cells (Tonbo Biosciences, CD28.2). Cells were stimulated for 72 hours at 37°C with 5% CO_2_ prior to downstream applications.

5-10x10^4^ PBMCs, unstimulated CD4+ T cells, and stimulated CD4+ T cells were taken for CD4 and CD25 cell surface marker staining (Miltenyi Biotec, #130-113-225, 1130-115-535, respectively) to assess cell population purity and activation for each donor. Stained samples were fixed in 1% formaldehyde in DPBS for analysis by flow cytometry within 1 week post-fixation.

### CRISPR-Cas9 Knock-Outs in Primary CD4+ T cells

CRISPR-Cas9 knock-outs were generated following previously published protocols [50]. To synthesize CRISPR-Cas9 ribonucleoprotein complexes (crRNPs), lyophilized CRISPR RNA (crRNA) and trans-activating CRISPR RNA (tracrRNA) (Dharmacon) were spun down and resuspended at 160 µM in buffer of 10 mM Tris-HCl (7.4 pH) and 150 mM KCl. For bulk crRNP synthesis, 10 µL of 160 µM crRNA was mixed with 10 µL of 160 uM tracrRNA, then incubated for 30 minutes at 37°C. The resulting crRNA:tracrRNA complexes were gently mixed with 20 µL of 40 µM *S. Pyogenes* Cas9 (UC-Berkeley Macrolab) then incubated at 37°C for 15 minutes. crRNPs were aliquoted into ten sets of 3.5 µL each and stored at -80°C. For pooled guide conditions, 5 independent crRNA targeting the same gene were mixed in equal volumes prior to tracrRNA addition. crRNAs were obtained from the Dharmacon predesigned Edit-R library or as custom sequences (see table of sequences below).[Table ppat.1013745.t002]

**Table ppat.1013745.t002:** 

Gene Target	Sequence	Catalog Number (Dharmacon)
NT #3	n/a	U-007503-20
NT #4	n/a	U-007504-20
CPSF6 #5	GGACCACATAGACATTTACG	CM-012334-05
CPSF6 #6	ATATATTGGAAATCTAACAT	Custom Sequence
CPSF5 #3	CAGGTTGATGGTGCGCTCCA	CM-012335-03
CPSF5 #12	CATGTGTTACTGCTGCAGCT	Custom Sequence
TRIM5α #6	AAGAAGTCCATGCTAGACAA	Custom Sequence
TRIM5α #7	GTTGATCATTGTGCACGCCA	Custom Sequence
CYPA #2	AGGTCCCAAAGACAGCAGGT	CM-004979-02
CYPA #3	GTACCCTTACCACTCAGTCT	CM-004979-03
CXCR4	GAAGCGTGATGACAAAGAGG	Custom Sequence
CCR5	AACACCAGTGAGTAGAGCGG	CM-004855-05
RPS2 #1–5 (Pool)	CGACCGACGCGTACCTTAAT,	CM-013690-01,
	TACCTTGCAAGGGACAGTGT,	CM-013690-02,
	CACACTGTCCCTTGCAAGGT,	CM-013690-03,
	AAGGCCGAGGATAAGGAGGT,	CM-013690-04,
	CCTACCGAAGTTGCCCAGGG	CM-013690-05
IFNAR1 #1–5 (Pool)	CGCCACGGCGACGAGCACTA,	CM-020209-01,
	AGTGTTATGTGGGCTTTGGA,	CM-020209–02,
	GCTCGTCGCCGTGGCGCCAT,	CM-020209–03,
	AGTGGATAATCCTGGATCAC,	CM-020209–04,
	GATCTAATGTTAAAGACTGG	CM-020209–05

To generate polyclonal knock-out cells, 1x10^6^ cells per electroporation reaction were spun down at 400xg for 5 minutes and supernatant was removed by aspiration. Cells were resuspended in electroporation buffer consisting of 16.4 µL P3 Nucleofector solution with 3.6 µL supplement per reaction. 20 µL of cell suspension was mixed with 3.5 µL of each preformed crRNP and transferred to 96-well electroporation cuvettes for electroporation with the 4D Core Unit using pulse code EH-115 (Lonza). 100 µL of warm cRPMI was added to each well following electroporation, and cuvettes were placed in cell culture incubator to recover at 37°C for 30 minutes. Cells were moved to 96-well flat bottom tissue culture plates prefilled with 100 µL complete RPMI, 2.5 µL T cell activation/expansion beads (Miltenyi Biotec, #130-091-441), and 0.2 µL of IL-2 (Miltenyi, #130097744). Cells were incubated at 37°C with 5% CO_2_.

### CRISPR-Cas9 Knock-In in HT1080 Cell Line

Endogenous monomeric NeonGreen (mNGreen) C-terminal tagged CPSF6 in HT1080 cell lines were generated by CRISPR-Cas9 knock-in. Briefly, antisense guide RNA (CACACATTTAACAGGGAACA) targeting sequences overlapping the stop codon of the CPSF6 gene locus was designed using the Benchling guide design online tool (Zang lab, MIT) and synthesized as lyophilized crRNA along with tracrRNA (IDT). The crRNA and tracrRNA were duplexed according to the manufacturer’s guidelines (IDT). 1 μM of the complexed sgRNA was mixed gently with 1µM of Alt-R *S. Pyogenes* Cas9 enzyme (IDT) in Opti-MEM (ThermoFisher, #11058021), incubated for 5 minutes at room temperature and stored in 4^o^C as an RNP complex. A template of mNGreen gene, flanked by 800 base pairs homology arms complementary to the CPSF6 loci, was PCR amplified and cloned into Adeno-associated virus (AAV) using the NheI and XhoI restriction sites in the AAV backbone (Cell Biolabs) flanked by the AAV-ITRs to generate the pAAV-CPSF6-mNGreen construct. AAV particles containing CPSF6-mNGreen template were produced as previously described [51]. Briefly, plasmids encoding the recombinant AAV DJ capsid and replication genes (pAAV-DJ Rep-Cap, Cell Biolabs), helper (pAAV-Helper, Cell Biolabs), and knock-in template (pAAV-CPSF6-mNGreen) were transfected in 293AAV cells in a 1:1:1 ratio using 1 mg/mL polyethylenimine (PEI, Polysciences, #23966). 72 hours post-transfection, the viral particles were harvested, filtered, aliquoted, and stored at -80^o^C. The sgRNA-Cas9 RNP complex was reverse transfected into 4x10^4^ HT1080 cells using RNAiMAX (ThermoFisher, #13778150) in a 96 well plate format and infected with 100 µL of AAV-containing CPSF6-mNGreen template. 48 hours post-transfection, cells were sub-cultivated in 48 well plate and sorted for single cell clones. Homozygous and heterozygous fluorescent knock-in CPSF6 cell clones were validated by PCR, sanger sequencing, western blotting and fluorescent widefield microscopy.

### Virus Stock Preparation

Replication competent HIV-1 was prepared using the original CXCR4-tropic HIV-1 NL4-3 nef:IRES:GFP plasmid (BEI Resources, #ARP-11349) in addition to the N74D and P90A capsid mutant plasmids described in the plasmid construction methods. Additional replication-competent virus was generated using the CCR5-tropic JR-FL iGFP plasmid and the transmitted founder CH040 plasmid (BEI Resources, #HRP-11740). For single round virus, a wild-type or N74D capsid mutant HIV NL4-3 dEnv nef:IRES:GFP plasmid was pseudotyped with VSV-G. For virus production, low passage (<15) HEK293T cells were plated at a density of 5x10^6^ cells per 15 cm tissue culture dish 24 hours prior to transfection. 10 µg of plasmid DNA, 500 µL serum-free DMEM, and 30 µL of PolyJet (SignaGen Laboratories, # SL100688) were combined per plate of virus and transfected according to manufacturer protocol. 25 mL of viral supernatant was collected at 48 hours post-transfection, replaced with fresh media, and a second set of supernatants were collected at 72 hours post-transfection. Media was combined, filtered through 0.2 µM filters (Fisherbrand, #FB12566504), and incubated with 8.5% polyethylene glycol (Sigma Aldrich, #81260–5 KG) and 0.3 M sodium chloride for 8 hours at 4°C. Viral precipitant was centrifuged at 3500 rpm for 20 minutes, then resuspended in 250 µL DPBS resulting in 100x concentration. Virus aliquots were stored at -80°C prior to p24 viral titer quantification and use in infection assays.

### p24 ELISA for Viral Titer Quantification

Viral stocks were heat inactivated at 60°C for 30 minutes, then quantified using an HIV-1 Gag p24 Quantikine ELISA kit (R&D Systems, #DHP240B) according to manufacturer protocol. Samples were run in duplicate at dilutions of 1:500 and 1:2500. Viral stock concentrations were determined using a standard curve.

### HIV-1 Spreading Infection Assays

CRISPR-Cas9 knock-out primary CD4+ T cells were generated as described above. All experiments included knock-outs for HIV-1 co-receptor *CXCR4*, host factor *CYPA*, and NT controls alongside knock-out of other host factors of interest. At day 4 post-electroporation, cells were split into replica plates of 1x10^5^ cells per well to generate samples for protein lysates for knock-out validation, viability assays, genomic DNA, HIV-1 infected protein lysates, and HIV-1 infection in triplicate per condition. Cells designated for protein lysates and viability assays were processed as described below. Genomic DNA was extracted using QuickExtract DNA Extraction Solution (Lucigen, #QE09050) according to manufacturer protocols and stored at -20°C. Plates designated for infection were infected in triplicate per condition with HIV-1 NL4-3 nef:IRES:GFP, JR-FL iGFP, or CH040 viral stocks diluted to a concentration of 0.2 ng p24 per 50 µL of complete RPMI with IL-2, and 50 µL of diluted virus was added to each well. Proper viral dose was determined by virus titration to optimize percent infection and cell viability (see S1e Fig). Infected cells were incubated in a cell culture incubator at 37°C with 5% CO_2_. 24 hours post infection, half of the cells received 50 µL of 25 µM Saquinavir (BEI Resources, #NIH-ARP 4658) diluted in complete RPMI with IL-2. The remaining cells received an equal volume of dimethyl sulfoxide (DMSO) (Sigma Aldrich, #472301–100ML) diluted in media. At days 2 and 5 post-infection, 75 µL of infected cell suspension was removed per well and fixed with 75 µL of 2% formaldehyde in DPBS for later analysis of infection by flow cytometry within 1 week post-fixation. For CH040-infecteted wells, cells were stained with anti-HIV-1 KC57 antibody and fixed according to the intracellular staining protocol below. The remaining cells were fed with an additional 75 µL of complete RPMI supplemented with IL-2. Protein lysates were collected from the appropriate plate on day 5 post-infection.

For spreading infections conducted in the presence of cyclosporin A, cells were treated with cyclosporin A (Sigma-Aldrich, #SML1018) at a final concentration of 5 µM 4 hours prior to infection.

### Single Round Infection Assays

CRISPR-Cas9 knock-out primary CD4+ T cells were generated as described above using cells from 3 independent blood donors. All experiments included knock-outs for HIV-1 co-receptor *CXCR4*, host factor *CYPA*, and NT controls alongside knock-out of other host factors of interest. At day 4 post-electroporation, cells were split into replica plates of 1x10^5^ cells per well to generate samples for protein lysates for knock-out validation, viability assays, and HIV-1 infection in triplicate per condition. Cells designated for protein lysates and viability assays were processed as described below. Plates designated for infection were infected in triplicate per condition with viral stocks (HIV-1 NL4-3 nef:IRES:GFP or VSV-G-pseudotyped NL4-3 dEnv nef:IRES:GFP) at a concentration of 0.02, 0.05, 0.1, 0.2, 0.5, or 1 ng p24 per well suspended in 50 µL complete RPMI supplemented with IL-2. At day 2 post infection, 75 µL of cell suspension was fixed with an equal volume of 2% formaldehyde in DPBS for later analysis of infection by flow cytometry within 1 week post-fixation.

### Interferon Titration and Sensitivity Assays

Primary CD4+ T cells from 3 independent donors underwent CRISPR-Cas9 editing using a NT control and guides targeting *CXCR4*, *CYPA*, *IFNAR1*, and *CPSF6*. Cells were replica plated as previously described. Additional replicates of 1x10^5^ edited cells per well in 96 well U-bottom plates were generated for the interferon titration assay. Plates for HIV-1 infection were treated with 100 U/mL (Donors P, Q, and AN) universal type I interferon (PBL Assay Science, #11200–2), IFNα (PBL Assay Science, #11100–1), IFNβ (PeproTech, #300–02BC), or IFNγ (PeproTech, #300–02) or a media-only control 16 hours prior to infection. Donor AO was treated with a range (1–1000 U/mL) of IFNα and IFNβ for 16 hours prior to infection. Following incubation with interferon, cells were infected with HIV-1 NL4-3 nef:IRES:GFP as described in spreading infection assay protocol. Infection timepoints were collected at days 2 and 5 post-infection, and day 5 HIV-1 infected protein lysates were collected as described below. To assess the impact of IFN treatment on ISG production, replica plated cells were treated with 10 U/mL of universal type I interferon, IFNα, IFNβ, IFNγ, or media-only controls. 16 hours post-interferon treatment, protein lysates were harvested according to protocol in immunoblotting methods for later analysis of ISG expression.

### Receptor/Co-receptor and Activation Marker Surface Staining

*CXCR4*, *CCR5*, *CYPA*, *CPSF6*, and *TRIM5α* knock-out cells and NT control cells were generated using CRISPR-Cas9 editing as previously described using primary CD4+ T cells isolated from the blood of three independent human donors. 1x10^5^ cells per condition were used for cell surface marker staining for CXCR4 (APC, Miltenyi Biotec, #130-120-708 [clone REA649]), CD4 (PE, Miltenyi Biotec, #130-113-225 [clone REA623]), CD25 (APC, Miltenyi Biotec, #130-115-535 [clone REA945]), and CCR5 (APC, Miltenyi Biotec, #130-120-708 [clone REA245]) at day 5 post-editing. Antibodies were diluted in MACS buffer at a dilution of 1:50 and samples were stained for 15 minutes at 4°C in the dark. After washing with MACS buffer, samples were fixed in 1% formaldehyde in DPBS for analysis by flow cytometry within 1 week post-fixation. Flow analysis was performed using the gating strategy shown in S7 Fig.

### Anti-HIV-1 Intracellular Staining

At days 2 and 5 post-infection, 75 µL of infected cell suspension from cells infected with virus lacking a fluorescent reporter (CH040) was removed per well and transferred to a new 96-well U-bottom plate. Cells were spun down at 400xg for 5 minutes, then the supernatant was aspirated and cells were fixed with 250 µL of 1% formaldehyde in DPBS for 30 minutes at room temperature. The fixed cells were spun down at 400xg for 5 minutes, the supernatant was aspirated, and then the cells were resuspended in 100 µL of a permeabilization buffer consisting of 1% BSA and 0.1% saponin (Sigma, #47036) in DPBS per well. Cells were blocked by incubating the cells in the permeabilization buffer for 20 minutes at room temperature. After blocking, the cells were spun down, the supernatant was aspirated, and the cells were stained with 100 µL of antibody solution consisting of a 1:100 dilution of anti-HIV-1 KC57-FITC antibody (Beckman Coulter, #6604665) in permeabilization buffer. Cells were incubated for 30 minutes in the dark at room temperature for staining. Cells were then spun down, washed with 200 µL of DPBS with 1% BSA, then fixed with 150 µL of 1% formaldehyde in DPBS for later analysis of infection by flow cytometry within 1 week post-fixation.

### Viability Assays

Cell viability was assessed at specified timepoints by Ghost Red amine dye stain and/or CellTiter-Glo assay. For Ghost Red stain, 1x10^5^ cells per condition were pelleted in 96-well U-bottom plates by centrifuging at 400xg for 5 minutes. Following a wash with DPBS, cells were resuspended in 100 µL of Ghost Red dye (Tonbo Biosciences, #13–0871-T100) diluted 1:1000 in DPBS. Cells were incubated in the dark at 4°C for 30 minutes, then spun down and supernatant was aspirated. Cells were washed with MACS buffer (DPBS (Corning, #21–031-CV) with 0.5% BSA (Fisher Scientific, BP9706100) and 2mM EDTA (Corning, #46–034-Cl)), spun down, and resuspended in 150 µL 1% formaldehyde in PBS for later analysis by flow cytometry.

For CellTiter-Glo Assay, cells in 96 well U-bottom plates were resuspended in 100 µL media and moved to an opaque, white 96 well plate (Corning, #3912). Cells were incubated at room temperature for 30 minutes to equilibrate to room temperature. Cell suspension was mixed with 100 µL of room temperature CellTiter-Glo reagent (Promega, #G7570) and placed on an orbital shaker for 2 minutes to complete cell lysis. Plate was incubated 10 additional minutes at room temperature to stabilize luminescence signal before reading luminescence on an Omega plate reader (BMG LabTech), with a consistent gain setting used across readings.

### Immunoblotting

Primary CD4+ T cell protein lysates were generated by washing 1x10^5^ cells per condition in DPBS, removing supernatant, and resuspending cells in 50 µL 2.5x Laemmli Sample Buffer (1.9 mL 0.5 M Tris-HCl pH 6.8 (Fisher Bioreagents, #BP153-1), 6 mL 50% glycerol (Fisher Bioreagents, #BP229-1), 3 mL 10% SDS (Corning, #46–040-CI), 250 µL β-mercaptoethanol (Fisher Chemical #O3666I-100), 50 µL 1% bromophenol blue (Fisher Bioreagents, #BP115–25), 18.8 mL DPBS (Corning, #21–031-CV)). Lysates were heated at 98°C for 20 minutes, then stored at -20°C prior to use for immunoblotting. Lysates were run on 4-20% Criterion Tris-HCl 18 or 26 well gels (Bio-Rad, #3450033 and # 3450034, respectively) at 90V for 30 minutes followed by 150V for 70 minutes. Proteins were transferred to PVDF membranes (Bio-Rad, #1620177) for 2 hours at 90V. Membranes were blocked in 4% milk in PBS (Sigma-Aldrich, #P5493-4L) with 0.1% Tween-20 (Fisher Bioreagents, #BP337–500) or 5% BSA in PBS with 0.1% Tween-20 according to antibody manufacturer recommendations for 1 hour, then incubated with primary antibody overnight at 4°C with shaking. Primary antibodies used in this study are as follows: CPSF6 (Rabbit, 1:3000 in 5% BSA, Novus, #NBP1–85676), CPSF5 (Rabbit, 1:5000 in 4% milk, Proteintech, #10322–1-AP), CYPA (Rabbit, 1:12000 in 4% milk, Cell Signaling, #2175), IFNAR1 (Rabbit, 1:1000 in 4% milk, abcam, #ab124764 [clone EPR6244]), MX1 (Rabbit, 1:1000 in 5% BSA, Cell Signaling, #37849S [clone D3W7I]), IFIT1 (Rabbit, 1:1000 in 4 milk, Cell Signalling #14769 [clone D2X9Z], TRIM5α (1:1000 in 4% milk, Cell Signaling, #14326 [clone D6Z8L]), and β-actin (Mouse, 1:10000 in 4% milk, Cell Signaling, #3700S [clone 8H10D10]). Membranes were washed with PBS with 0.1% Tween-20, then incubated with either anti-mouse (Peroxidase AffiniPure Goat Anti-Mouse IgG (H + L), Jackson ImmunoResearch, #115-035-003) or anti-rabbit (Peroxidase AffiniPure Goat Anti-Rabbit IgG (H + L), Jackson ImmunoResearch, # 111-035-003) HRP-conjugated secondary antibodies at 1:10000 in 4% milk in PBS with 0.1% Tween-20 for 1 hour, washed with PBS with 0.1% Tween-20, then detected using Immobilon Western Chemiluminescent HRP Substrate (EMD Millipore, #WBKLS0500) and imaged using an iBright imaging system (Thermo Fisher). Blots were incubated in antibody stripping solution (EMD Millipore, #2502) prior to re-probing. Blots were quantified by measuring the rolling ball-corrected density of each analyzed band using iBright Image Analysis Software (version 5.2.1.0). Density values for the target protein bands (CPSF6 or IFIT1) were divided by density values for β-actin loading control bands, and the resulting ratio was normalized to the NT control ratio for each donor.

### Flow Cytometry

Flow cytometry analysis was performed using an Attune NxT acoustic focusing cytometer and CytKick Max Autosampler attachment (Thermo Fisher Scientific). Samples were suspended in 150 µL DPBS with 1% formaldehyde and were subjected to one 150 µL mixing cycle. All events in 50 µL of sample were recorded. FCS3.0 files were exported using Attune NxT Software (version 5.3.0) and analyzed using a FlowJo (version 10.10) template shown in S7 Fig for HIV-1 infection (BL1:GFP and FITC), Ghost Red stain (RL-2), or cell surface receptor stain (RL-1: CXCR4-APC, CCR5-APC, CD25-APC, BL-2: CD4-PE).

### HIV-1 Infection and Live Cell Sorting for RNA-Seq

21x10^6^ stimulated primary CD4 + T cells from donors AC-AE were infected with wild-type or N74D capsid mutant HIV-1 NL4–3 nef:IRES:GFP alongside uninfected controls. Briefly, cells were resuspended in complete RPMI supplemented with IL-2, concentrated HIV-1 stocks for the appropriate virus were added to the cell suspension at a dose of 210 ng per condition, and the final volume of media was brought to 42 mL. Cells were then distributed into 96 well U-bottom plates, with each well containing 200 µL media, 1x10^5^ cells, and 1 ng p24. 1x10^6^ cells per donor were distributed into 96 well U-bottom plates at a concentration of 1x10^5^ cells per well in 200 µL complete RPMI with IL-2 to generate uninfected control cells. All cells were incubated in a cell culture incubator at 37°C with 5% CO_2_. On day 2 post-infection, infected cells were pooled per donor and virus strain. 1x10^6^ uninfected cells per donor immediately underwent RNA extraction according to protocol below to generate the uninfected RNA samples. The remaining infected cells (~20 million per condition) were spun down at 400xg for 5 minutes, resuspended in 1 mL MACS buffer, and transferred to MACSQuant Tyto Cartridges (Miltenyi Biotec, #130-104-791). Cells in cartridges were immediately sorted using a MACSQuant Tyto sorter (Miltenyi Biotec) with MACSQuantify Tyto Software (version 2.0). Cells were sorted by gating for infection (GFP+) status as described in S7 Fig The cartridge mixer was set at a speed of 800 rpm, and the cell sorting pressure was set to 150 hPa. Following sorting, cells were resuspended in 200 µL MACS buffer and retrieved from the sorted cartridge chamber. Samples were spun at 400xg for 5 minutes, supernatant was aspirated, and RNA was extracted according to the protocol below.

### RNA Extraction

RNA was extracted from donors E-G (Fig 2) at day 5 post electroporation and donors AC-AE (Fig 6) at day 2 post-infection or day 2 post-electroporation depending on the condition. RNA was extracted from cell pellets using RNeasy Mini Kit (QIAGEN, #74104) according to manufacturer protocol. Samples were treated with DNAse I according to manufacturer protocol (QIAGEN, # 79254) and eluted in RNase-free water. RNA concentration was determined by NanoDrop (Thermo Scientific), and samples were stored at -80°C prior to analysis.

### HT1080-CPSF6-mNGreen HIV-1 Infection, RNA Extraction, Immunofluorescence Staining, and Imaging

1x10^5^ HT1080-CPSF6-mNGreen cells per condition were seeded in 12-well plate and adhered to plates overnight prior to infection. In parallel, 3x10^4^ HT1080-CPSF6-mNGreen cells were seeded in 8-well chamber slides (Ibidi, #80806) coated with 0.1 mg/ml poly-D lysine hydrobromide (Advanced Biomatrix, #5049). These cells were infected with VSV-G pseudotyped wild-type or N74D capsid mutant HIV-1-NL4-3 at an MOI of 10 or mock-infected by spinoculation at 16°C for 1 h at 1200xg. Cell cultures were washed once with prewarmed DMEM and returned to a tissue culture incubator at 37°C with 5% CO_2_ for 6 hours. RNA from wild type, N74D capsid mutant HIV-1-NL4-3, and mock infected cells in the 12-well plate was then extracted using a NucleoSpin RNA Mini kit (Machery-Nagel, # 740955.50) according to manufacturer’s protocol. RNA concentration was determined by NanoDrop (Thermo Scientific), and samples were stored at -80°C prior to analysis. Protein lysates were harvested from HIV-1-NL4-3 wild-type, N74D and mock infected HT1080-CPSF6-mNGreen cells infected under the same conditions described above for analysis of CPSF6 protein levels by immunoblot. In parallel, HIV-1-NL4-3 wild-type, N74D and mock infected HT1080-CPSF6-mNGreen cells cells on the 8-well chamber slides were washed with 1x DPBS and fixed with 4% paraformaldehyde (PFA) (Sigma, #P6148-1 KG) for 15 minutes. The PFA fixation was quenched with 5mM glycine for 10 minutes and washed twice with 1x DPBS. The cells were permeabilized and blocked with 0.1% Triton- X100 and 10% goat serum respectively for 30 minutes. Mouse monoclonal anti-SC35 antibody (abcam, #ab11826) was subsequently used to stain the cells at a 1:250 dilution for 1 hour, washed twice with 1x DPBS, followed by goat anti-mouse secondary antibody conjugated to janelia fluor 646 (Novus Biologicals, #72739JF646) at a 1:500 dilution for 1 hour. Mock infected control cells were stained with purified mouse IgG1 kappa isotype control antibody (Biolegend, #401401) at a 1:250 dilution. Following the secondary antibody staining, the cells were washed twice and stained with Hoechst 33258 (abcam, #ab145596) for DNA at a 1:1000 dilution for 30 minutes. Images for each experimental condition were acquired with widefield deconvolution fluorescent microscope (GE, DeltaVision OMX SR Imaging System) equipped with 60x objective with 1.42 NA, using GE oil emersion with 1.518 refractive index, complementary metal-oxide semiconductor (CMOS) cameras and 405 nm, 488 nm, 568 nm and 640 nm Diode lasers. Acquisitions were performed with the following conditions, 10% transmission and 100-ms exposure for the green channel (CPSF6), 10% laser power and 100ms exposure for the far-red channel (SC35), and 5% laser power and 100ms exposure for the blue channel (Hoechst 33258). All images were acquired with the same microscope settings and deconvolved using the ratio conservative algorithm method SoftWorx (version 7.2.2).

### Quantification of CPSF6 Puncta and CPSF6/SC35 Colocalization

Images in the CPSF6, SC35 and Hoechst channels were acquired deconvoluted as described above. Images were then opened in Imaris (version 10.2) and CPSF6 puncta were detected using the Imaris Spots module (estimated XY diameter 0.25 µm, estimated Z diameter 0.5 µm, quality > 300, region growing option was used to account for varying spot sizes). SC35 nuclear speckles were segmented using the Imaris Surfaces module (surface grain size = 0.1 um, manual threshold = 3000). The detected CPSF6 puncta were then classified into colocalizing (class A) and non-colocalizing (class B) with SC35. An Imaris pipeline was created with the above parameters and applied to all images in the batch. The sum fluorescence intensity of each spot in conditions (Mock, WT, and N74D) was exported as a.csv file.

### RNA-Sequencing

RNA samples from donors E-G (Fig 2) were submitted to the NUSeq core for next-generation sequencing. Libraries were prepared using TruSeq Total RNA-Seq Library Prep (Illumina) and sequenced using an Illumina NovaSeq 6000 with 150 bp paired-end reads. RNA samples from donors AC-AE (Fig 6) were submitted to Novogene for next-generation sequencing. Libraries were prepared using NEBNext Ultra II kit with poly(A) selection according to manufacturer protocols (New England Biolabs). Samples were sequenced using an Illumina NovaSeq 6000 with 150 bp paired-end reads. RNA samples from HT1080 cells (Fig 6) were submitted to Novogene for next-generation sequencing. Libraries were prepared using NEBNext Ultra II kit with poly(A) selection according to manufacturer protocols (New England Biolabs). Samples were sequenced using a NovaSeq X Plus with 150 bp paired-end reads.

### RNA-Sequencing Data Processing

The RNA-Seq data processing pipeline was adapted from published protocols [52]. Briefly, we first trimmed the paired-end reads in FASTQ files using Trimmomatic (version 0.39) [53], targeting known adapters (TruSeq3-SE.fa:2:30:10), and also trimming the first and last 3 base pairs if their quality fell below set thresholds. Additionally, we employed a 4-base sliding window technique, trimming reads when the average window quality dropped below 15. Reads shorter than 36 base pairs were excluded. Next, the trimmed reads were aligned to the human reference genome GRCh38 (https://www.ncbi.nlm.nih.gov/datasets/genome/GCF_000001405.26/) using HISAT2 [54] (version 2.1.0) and converted into BAM format using samtools (version 1.6) [55]. The alignment data was then processed by StringTie (version 2.1.3) [56] to estimate gene expression levels. The StringTie merge function merged samples’ gene structures to create a unified set of transcripts across all samples. Finally, read counts were obtained using prepDE.py (https://ccb.jhu.edu/software/stringtie/index.shtml?t=manual) to prepare the data for DESeq2.

### Differential Gene Expression Analysis

The raw read counts were processed in R (version 4.2.3). Initially, transcript names were converted to gene symbols, eliminating duplicate symbols and dropping transcripts that did not map to known gene symbols. The resulting reads were used to construct a DESeqDataSetFromMatrix object using DESeq2 (version 1.38.3) [57] for investigating differences across conditions, accounting for donor variability. Genes with a minimum count of 10 in at least 3 samples were retained for analysis. To visualize treatment effects, counts were transformed using the variance stabilizing transformation (VST) function from DESeq2 and subjected to principal components analysis (PCA), visualizing the first two principal components [58].

Differential gene expression was assessed using the DESeq function from DESeq2, which estimates size factors and dispersions, fitting a negative binomial generalized linear model. P-values were adjusted using the Benjamini-Hochberg method [59], considering adjusted p-values below 0.05 as significant. Subsequently, complete cases were filtered, and the lists of differentially expressed genes were input into Metascape (version 3.5.20250101) for identifying pathways associated with up- or downregulated gene sets [60]. The log fold change for genes identified by Metascape that were associated with the interferon gamma signaling pathway were visualized for all 3 donors (individually and collectively) in a heatmap. The top 2000 significant genes with highest log fold changes were also z-score transformed and clustered using hierarchical clustering that was visualized in a heatmap [61].

### Alternative Polyadenylation Analysis

APA analysis was conducted by analyzing RNA-Seq data using the REPAC pipeline (version 0.99.0) to determine changes in 3’ UTR length in treated (*CPSF6* knock-out or infected) versus control (non-targeting or uninfected) conditions [36]. Polyadenylation sites that had less than 20 counts assigned to them were excluded from further analysis. Differential polyadenylation site usage was then calculated between experimental (*CPSF6* knock-out or HIV-1 infected) versus control (non-targeting or uninfected) conditions. 3’ UTRs were designated shortened for Compositional Fold Change (cFC) < -0.25 and -log_10_(adjusted p-value) < 0.05 and lengthened for cFC > 0.25 and -log_10_(adjusted p-value) < 0.05. Lists of genes with significant changes in 3’ UTR length were input into Metascape (version 3.5.20250101) for identifying pathways associated with genes sets with shortened or lengthened 3’ UTRs, and the list of all genes with tested APA sites was provided as a background gene set [60].

Additional validation of 3’ UTR length changes were carried out using DaPars (Dynamic analysis of Alternative PolyAdenylation from RNA-Seq) (version 0.9.1) [37,62]. Distal polyadenylation sites (PAS) for each gene were extracted from the GRCh38/hg38 refseq gene model downloaded from UCSC (https://genome.ucsc.edu/). A proximal PAS for each gene was determined using DaPars by identifying the optimal fitting point of a linear regression model using alignment wiggle files for each sample created using DeepTools (version 3.5.6). Differential PAS usage was then quantified as a change in Percentage of Distal PAS Usage Index (ΔPDUI) for each gene in treated (*CPSF6* knock-out or infected) versus control (non-targeting or uninfected) conditions, using default parameters. Genes were considered significantly shortened or lengthened if they had a false discovery rate (FDR) of < 0.05.

RNA-Seq read tracks were visualized using Integrated Genomics Viewer (version 2.17.4) to show changes in 3’ UTR read coverage for genes of interest (*TGFBR1*, *IL2RA*/*CD25*, *CXCR4*) using RNA-Seq data from Donor E. Track heights were kept consistent across samples. Violin plots depicting changes in 3’ UTR length among gene sets of interest (Cytokine Signaling in Immune system (R-HSA-1280215), Interferon signaling (R-HSA-913531), and T cell activation (GO:0042110)) were generated by extracting gene names associated with the gene sets using biomaRt (version 2.58.2) and annotating the output of the REPAC and ΔPDUI analyses to indicate which of the tested genes were a member of the gene sets.

### Statistics

All statistical analysis was performed using GraphPad Prism version 10.2.1 (395) for Windows, GraphPad Software, Boston, Massachusetts USA, www.graphpad.com and R (version 4.2.3) stats package (version 3.6.2). All statistical analysis performed in this manuscript is available in S1 Table.

## Supporting information

S1 Fig Additional validation of HIV-1 infection phenotype in *CPSF6* knock-out primary CD4+ T cells. a, Immunoblot shows knock-out of *CPSF6* and *CYPA* in primary CD4+ T cell protein lysates harvested at day 4 post-editing in 3 biological replicates (donors A, B, and D). b, Immunoblot shows knock-out of *CPSF6* in primary CD4+ T cell protein lysates harvested at days 1-10 post-editing in 1 representative biological replicate (donor AF). C, Knock-out primary CD4 + T cells exhibit similar viability to NT controls up to day 10 post nucleofection as assessed by Ghost Red amine dye stain (top) or CellTiter Glo viability assay (bottom) in 3 biological replicates (donors AG-AI). Ribosomal protein S2 (*RPS2*) knock-out serves as a control that is lethal in primary CD4+ T cells. Dots in Ghost Red charts represent cell count in the Ghost Red- gate (number of viable cells) at the indicated timepoint. Dots in Cell Titer Glo charts represent luminescence signal at the indicated timepoint. d, HIV-1 infectivity (Raw % GFP positive cells) at days 2 (left) and 5 (right) post-challenge with HIV-1 NL4-3 nef:IRES:GFP in indicated knock-out primary CD4+ T cells from 4 biological replicates (donors A-D) as assessed by flow cytometry. Cells were treated with protease inhibitor (saquinavir) or DMSO control at 24 hours post-challenge as indicated. Each bar represents the average of technical triplicates + /- SD with individual data points shown. Statistics were calculated relative to the NT control per condition by one-way ANOVA with Dunnet’s test for multiple comparisons; * = p ≤ 0.05, ** = p ≤ 0.01, *** = p ≤ 0.001, **** = p ≤ 0.0001. e, HIV-1 infectivity (% GFP positive cells normalized to NT control per donor) at day 5 post-challenge with HIV-1 NL4-3 nef:IRES:GFP in indicated knock-out primary CD4+ T cells from 4 biological replicates (donors AJ-AM) as assessed by flow cytometry. Cells were infected with a range of HIV-1 NL4-3 nef:IRES:GFP concentrations standardized by p24. Each bar represents the average of technical triplicates + /- SD with individual data points shown. Statistics were calculated relative to the NT control per condition by one-way ANOVA with Dunnet’s test for multiple comparisons; * = p ≤ 0.05, ** = p ≤ 0.01, *** = p ≤ 0.001, **** = p ≤ 0.0001.
(TIF)

S2 Fig *CPSF6* knock-out has broad transcriptional impact in primary CD4+ T cells. a, Immunoblot shows knock-out of *CPSF6* and *TRIM5α* in primary CD4+ T cell protein lysates harvested at day 5 post-editing in 2 biological replicates (donor F and G). b, Knock-out primary CD4+ T cells exhibit similar viability to NT controls at day 5 post-editing as assessed via amine dye stain and flow cytometry. Dots represent cell viability (% Ghost Red negative cells) per condition, horizontal lines represent the average of viability measurements in 3 biological replicates (donors E-G). Statistics were calculated relative to the NT control by two-way ANOVA with Dunnet’s test for multiple comparisons; ns = not significant. c, Principal component analysis (PCA) of RNA-Seq data from *CPSF6* knock-out, *TRIM5α* knock-out, and NT controls in 3 biological replicates (donors E-G). d, Heatmap shows normalized z-score for each of the top 2000 differentially expressed genes as ranked by absolute value of log_2_(fold change) in *CPSF6* knock-out, *TRIM5α* knock-out, and NT controls cells in 3 biological replicates (donors E-G). e, Heatmap shows differential expression (log_2_(fold change)) of genes in *CPSF6* knock-out primary CD4+ T cells as compared to NT controls in the Reactome term “Interferon Signaling” (R-HSA-913531) in 3 biological replicates (donors E-G). f, Schematic shows differential gene expression (log_2_(fold change)) for 3 biological replicates (donors E-G) in *CPSF6* knock-out primary CD4+ T cells as compared to NT controls overlayed on schematic of signaling events downstream of the type I and type II interferon receptors, including ISGs that are induced by these signaling pathways. g, Chart shows TRIM5α band density divided by β-actin band density in knock-out validation blot for donor E (Fig 2b). Density measurements are normalized to NT control. h, Graphs show mean fluorescence intensity (MFI) of CD4 (first), CXCR4(second) CCR5 (third) and CD25 (fourth) in primary CD4 + T cells from 3 biological replicates (donors H-J) at day 5 post-editing as measured by immunostaining and flow cytometry. Dots represent MFI per condition, horizontal lines represent the average of MFI measurements in 3 biological replicates. Statistics were calculated relative to the NT control by two-way ANOVA with Dunnet’s test for multiple comparisons; ns = not significant, * = p ≤ 0.05, ** = p ≤ 0.01, *** = p ≤ 0.001, **** = p ≤ 0.0001.
(TIF)

S3 Fig CPSF6-mediated regulation of the interferon pathway and TRIM5α expression is reproducible across multiple, independent donors. a, HIV-1 infectivity (Raw % GFP positive cells) at day 5 post-challenge with HIV-1 NL4-3 nef:IRES:GFP in indicated knock-out primary CD4+ T cells from 1 biological replicate (AN) as assessed by flow cytometry. Cells were pre-treated with 100 U/mL IFNα, IFNβ, IFNγ, Universal Type I IFN, or media only control. Each bar represents the average of technical triplicates + /- SD with individual data points shown. Statistics were calculated relative to the NT control per condition by one-way ANOVA with Dunnet’s test for multiple comparisons; * = p ≤ 0.05, ** = p ≤ 0.01, *** = p ≤ 0.001, **** = p ≤ 0.0001 b, HIV-1 infectivity (Raw % GFP positive cells) at day 5 post-challenge with HIV-1 NL4-3 nef:IRES:GFP (top) or VSV-G pseudotyped HIV-1 NL4-3nef:IRES:GFP (bottom) in indicated knock-out primary CD4+ T cells from 1 biological replicate (AO) as assessed by flow cytometry. Cells were pre-treated with indicated doses of IFNα (left) or IFNβ (right) for 16 hours prior to infection. Statistics are shown for CPSF6 #6 compared to the non-targeting (NT) control per condition, calculated by one-way ANOVA with Dunnet’s test for multiple comparisons; * = p ≤ 0.05, ** = p ≤ 0.01, *** = p ≤ 0.001, **** = p ≤ 0.0001, ns = not significant. c, Immunoblots (left) show expression of representative ISGs (IFIT1 and MX1) in knock-out primary CD4+ T cells treated with 10 U/mL IFNα, IFNβ, Universal Type I IFN, IFNγ, or media-only control for 16 hours in 2 biological replicates (donors Q and AN). Charts (right) show IFIT1 band density divided by β-actin band density in adjacent blots. Density measurements are normalized to NT controls per condition. d, Immunoblot shows knock-out of *CPSF6*, *TRIM5α*, and *CYPA* in primary CD4+ T cell protein lysates harvested at day 4 post-editing in 5 biological replicates (donors R-V). e, HIV-1 infectivity (Raw % GFP positive cells) at day 5 post-challenge with HIV-1 NL4-3 nef:IRES:GFP P90A capsid mutant in indicated knock-out primary CD4+ T cells from 5 biological replicates (donors R-V) as assessed by flow cytometry. Each bar represents the average of technical triplicates + /- SD with individual data points shown. Statistics were calculated relative to the NT control per condition by one-way ANOVA with Dunnet’s test for multiple comparisons; * = p ≤ 0.05, ** = p ≤ 0.01, *** = p ≤ 0.001, **** = p ≤ 0.0001.; * = p ≤ 0.05, ** = p ≤ 0.01, *** = p ≤ 0.001, **** = p ≤ 0.0001. f, Immunoblot shows knock-out of *CPSF6*, *TRIM5α*, and *CYPA* in primary CD4+ T cell protein lysates harvested at day 4 post-editing in 3 biological replicates (donors W-Y).
(TIF)

S4 Fig *CPSF6* knock-out primary CD4+ T cells exhibit shortening of 3’ UTRs a, Tracks show read coverage of IL2Ra/CD25 (top) and CXCR4 (bottom) in RNA-Seq data from *CPSF6* knock-out and NT control cells from one representative biological replicate (donor E), visualized using Integrative Genomics Visualizer. 3’ UTRs are annotated for each gene. b, Volcano plot shows DaPars analysis of changes in 3’ UTR lengths for *CPSF6* knock-out versus NT control primary CD4+ T cells from 3 biological replicates (donors E-G). 3’ UTRs are designated shortened (blue) for ΔPDUI < 0 and -log_10_(adjusted p-value) < 0.05 and lengthened (red) for ΔPDUI > 0 and -log_10_(adjusted p-value) < 0.05. Dots represent tested genes. c, Violin plot shows comparison of ΔPDUI values from DaPars analysis in all tested genes as compared to genes in the indicated gene sets (GO:0042110:T Cell activation, R-HSA-1280215: Cytokine Signaling in Immune system, R-HSA-913531: Interferon Signaling) in 3 biological replicates (donors E-G). d, *CPSF5* knock-out primary CD4+ T cells exhibit similar viability to NT controls at day 4 post-editing as assessed via amine dye stain and flow cytometry. Dots represent cell viability (% Ghost Red negative cells) per condition, horizontal lines represent the average of viability measurements in 3 biological replicates (donors Z-AB).
(TIF)

S5 Fig HIV-1 infection and *CPSF6* knock-out lead to broad transcriptional reprogramming in HT1080 cells. a, Widefield deconvolution fluorescent microscopy images of HT1080-CPSF6-mNGreen cells with immunofluorescent staining for a marker of nuclear speckles, SC35, or an IgG isotype control (top left set of images). Cells were mock infected (top 2 sets of images) or infected with VSV-G pseudotyped WT (bottom left set of images) or N74D capsid mutant (bottom right set of images) HIV-1-NL4-3 at an MOI of 10 at 6 hours post-infection. CPSF6-mNGreen shown in green, Hoechst DNA stain shown in blue, SC35 shown in red. 3 representative images shown per condition. b, Graph shows quantification of the intensity sum of fluorescent signal for CPSF6 + /SC35- and CPSF6 + /SC35 + puncta in HT1080-CPSF6-mNGreen cells mock infected or infected with VSV-G pseudotyped WT or N74D capsid mutant HIV-1-NL4–3 at an MOI of 10 depicted in panel a. CPSF6 puncta were detected and SC35 nuclear speckles were segmented using Imaris, and the detected CPSF6 puncta were then classified into either colocalizing or non-colocalizing with SC35. c, Immunoblot shows expression of CPSF6 in HT1080-CPSF6-mNGreen cells mock infected or infected with VSV-G pseudotyped WT or N74D capsid mutant HIV-1-NL4-3 at an MOI of 10 at 6 hours post-infection in 2 technical replicates.
(TIF)

S6 Fig HIV-1 infection and *CPSF6* knock-out lead to broad transcriptional reprogramming in primary CD4+ T cells a, Schematic shows experimental workflow for RNA-Seq of infected primary CD4+ T cells. Primary CD4+ T cells were isolated from the blood of three independent donors (donors AC-AE). One subset of cells was edited via electroporation of crRNPs targeting *CPSF6* alongside NT controls, and RNA was extracted for bulk RNA sequencing at day 2 post-editing. The other subsets of cells were infected with wild-type (WT), or N74D HIV-1 NL4-3 nef:IRES:GFP alongside uninfected controls. HIV-1 infected (GFP+) cells were sorted at day 2 post-infection and RNA was immediately extracted for bulk RNA sequencing. b, Immunoblot shows knock-out of *CPSF6* at day 2 post-editing in 3 biological replicates (donors AC-AE). c. Top volcano plot shows differentially expressed genes in RNA-Seq data from WT HIV-1 NL4-3 nef:IRES:GFP-infected primary CD4+ T cells as compared to uninfected controls in 3 biological replicates (donors AC-AE). Dots show significantly downregulated (blue) and upregulated (red) genes, dotted line shows threshold of 0.05 for adjusted p-value. Bottom volcano plot shows REPAC analysis of changes in 3’ UTR lengths for *CPSF6* knock-out primary CD4+ T cells as compared to NT controls in 3 biological replicates (donors AC-AE). 3’ UTRs are designated shortened (blue) for Compositional Fold Change (cFC) < -0.25 and -log_10_(adjusted p-value) < 0.05 and lengthened (red) for cFC > 0.25 and -log_10_(adjusted p-value) < 0.05. Dots represent individual altered 3’ UTRs. d, Charts show functional enrichment (Metascape) analysis of top 10 upregulated (left, red) and downregulated (right, blue) pathways in differentially expressed genes from *CPSF6* knock-out primary CD4+ T cells as compared to NT controls at day 2 post-editing in 3 biological replicates (donors AC-AE) e, Volcano plot shows differentially expressed genes in RNA-Seq data from WT capsid mutant HIV-1 NL4-3 nef:IRES:GFP-infected primary CD4+ T cells as compared to uninfected controls in 3 biological replicates (donors AC-AE). Dots show significantly downregulated (blue) and upregulated (red) genes, dotted line shows threshold of 0.05 for adjusted p-value. f, Volcano plot shows differentially expressed genes in RNA-Seq data from N74D capsid mutant HIV-1 NL4-3 nef:IRES:GFP-infected primary CD4+ T cells as compared to uninfected controls in 3 biological replicates (donors AC-AE). Dots show significantly downregulated (blue) and upregulated (red) genes, dotted line shows threshold of 0.05 for adjusted p-value. g, Venn diagrams shows overlap of upregulated (top) and downregulated (bottom) differentially expressed genes between *CPSF6* knock-out, WT infected, and N74D infected primary CD4+ T cells in 3 biological replicates (donors AC-AE). h, Charts show functional enrichment (Metascape) analysis of top 10 upregulated (left, red) and downregulated (right, blue) pathways in differentially expressed genes from WT HIV-1 NL4-3 infected primary CD4+ T cells that are not differentially expressed in N74D HIV-1 NL4-3 infected primary CD4+ T cells at day 2 post-infection in 3 biological replicates (donors AC-AE). i, List of individual genes that undergo 3’ UTR shortening upon WT HIV-1 NL4-3 infection from analysis shown in Fig 6g. j, Volcano plot shows DaPars analysis of changes in 3’ UTR lengths for WT HIV-1 NL4-3 nef:IRES:GFP infected versus uninfected primary CD4+ T cells from 3 biological replicates (donors AC-AE). 3’ UTRs are designated shortened (blue) for ΔPDUI < 0 and -log_10_(adjusted p-value) < 0.05 and lengthened (red) for ΔPDUI > 0 and -log_10_(adjusted p-value) < 0.05. Dots represent tested genes. k, Volcano plot shows DaPars analysis of changes in 3’ UTR lengths for N74D capsid mutant HIV-1 NL4-3 nef:IRES:GFP infected versus uninfected primary CD4+ T cells from 3 biological replicates (donors AC-AE). 3’ UTRs are designated shortened (blue) for ΔPDUI < 0 and -log_10_(adjusted p-value) < 0.05 and lengthened (red) for ΔPDUI > 0 and -log_10_(adjusted p-value) < 0.05. Dots represent tested genes. l, HIV-1 infectivity (% GFP positive cells normalized to NT control per donor) at day 5 post-challenge with HIV-1 NL4-3 nef:IRES:GFP N74D capsid mutant virus in indicated knock-out primary CD4+ T cells from 3 biological replicates (donors R-V) as assessed by flow cytometry. Each bar represents the average of technical triplicates + /- SD with individual data points shown. Statistics represent one-way ANOVA with Dunnet’s test for multiple comparisons, compared to NT within each condition; * = p ≤ 0.05, ** = p ≤ 0.01, *** = p ≤ 0.001, **** = p ≤ 0.0001.
(TIF)

S7 Fig Flow Cytometry Gating Strategy. a, Flow gating strategy for quantification of percent HIV-1 NL4–3 nef:IRES:GFP infected primary CD4 + T cells through sequential application of a lymphocyte gate, two single-cell gates, and GFP autofluorescence exclusion (FlowJo v10.10). For cells sorted using the MACSQuant Tyto sorter, cells were gated on backscatter (BSB) rather than forward scatter (FSC). b, Flow gating strategy for quantification of percent viable primary CD4+ T cells through sequential application of an all-cell gate, two single-cell gates, and a fluorophore gate (FlowJo v10.10). c, Flow gating strategy for quantification of fluorescence intensity of cell surface receptors through sequential application of a lymphocyte gate, two single-cell gates, and a histogram showing cell count versus fluorescence intensity measurement of the fluorophore of interest (FlowJo v10.10).
(TIF)

S1 Table Statistical Analysis Table. Excel table contains a full description of statistical tests performed in this study, with tabs corresponding to each figure.
(XLSX)

S2 Table Processed Primary CD4+ T cell RNA-Sequencing Data. Excel table contains full processed datasets for all primary CD4+ T cell RNA-Sequencing experiments performed in this study, with tabs corresponding to each figure.
(XLSX)

S3 Table Source Data. Excel file contains processed flow cytometry data. Each sheet in the Excel file corresponds to the indicated figure panel.
(XLSX)

S4 Table Uncropped Immunoblot Images for Main Text Figures. Excel file contains uncropped blot images for main text figures. Each sheet in the Excel file corresponds to the indicated figure panel.
(XLSX)

S5 Table Uncropped Immunoblot Images for Supplementary Figures. Excel file contains uncropped blot images for supplementary figures. Each sheet in the Excel file corresponds to the indicated figure panel.
(ZIP)
