# Supplementary figures and images for: Alternative polyadenylation upon CPSF6 knock-out enhances HIV-1 infection in primary T cells
Source: PLoS Pathog. 2025 Dec 12;21(12):e1013745. doi: 10.1371/journal.ppat.1013745 (PMC12714223; doi:10.1371/journal.ppat.1013745)

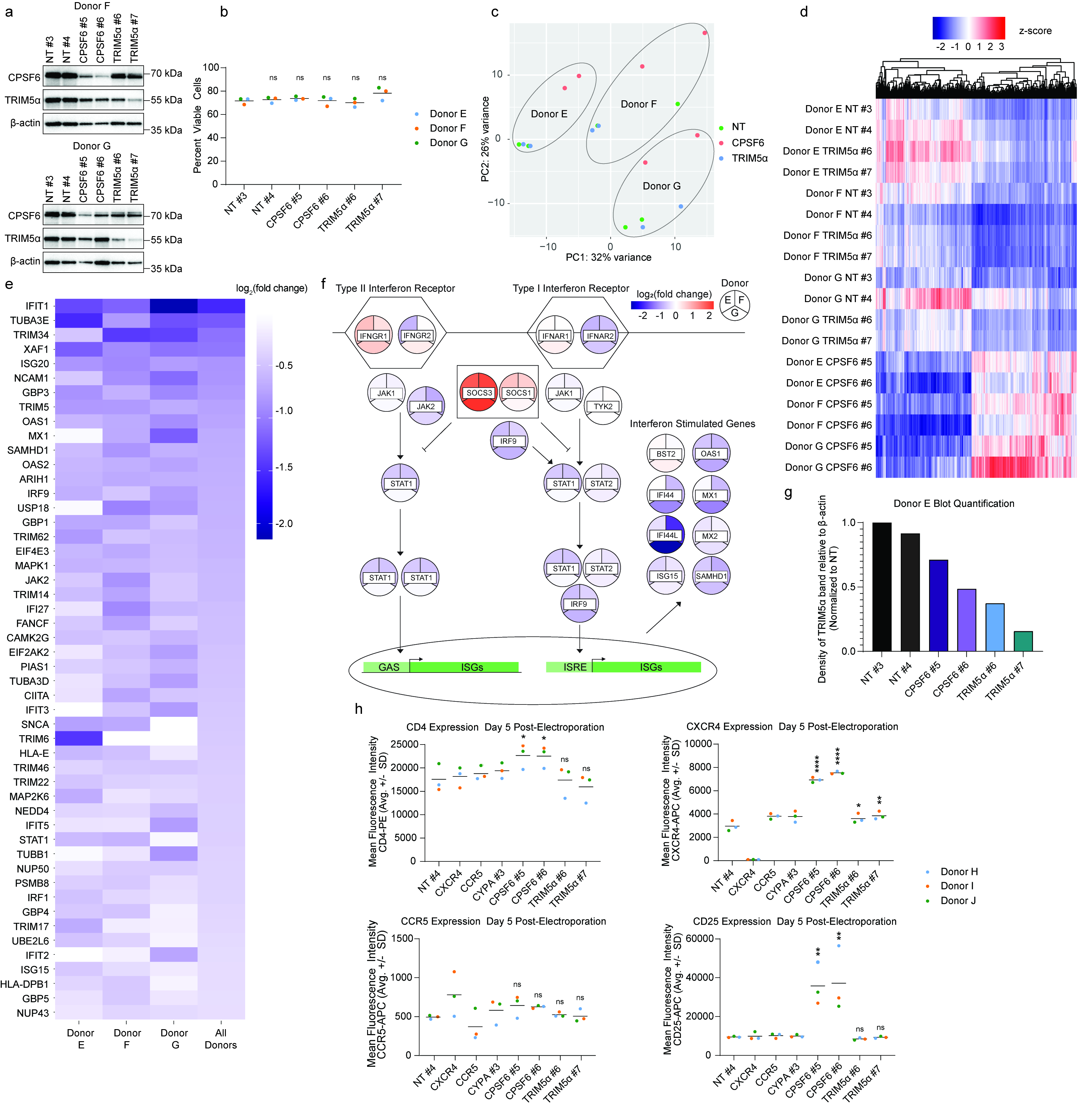

Supplement: S2 Fig — a, Immunoblot shows knock-out of CPSF6 and TRIM5α in primary CD4+ T cell protein lysates harvested at day 5 post-editing in 2 biological replicates (donor F and G). b, Knock-out primary CD4+ T cells exhibit similar viability to NT controls at day 5 post-editing as assessed via amine dye stain and flow cytometry. Dots represent cell viability (% Ghost Red negative cells) per condition, horizontal lines represent the average of viability measurements in 3 biological replicates (donors E-G). Statistics were calculated relative to the NT control by two-way ANOVA with Dunnet’s test for multiple comparisons; ns = not significant. c, Principal component analysis (PCA) of RNA-Seq data from CPSF6 knock-out, TRIM5α knock-out, and NT controls in 3 biological replicates (donors E-G). d, Heatmap shows normalized z-score for each of the top 2000 differentially expressed genes as ranked by absolute value of log2(fold change) in CPSF6 knock-out, TRIM5α knock-out, and NT controls cells in 3 biological replicates (donors E-G). e, Heatmap shows differential expression (log2(fold change)) of genes in CPSF6 knock-out primary CD4+ T cells as compared to NT controls in the Reactome term “Interferon Signaling” (R-HSA-913531) in 3 biological replicates (donors E-G). f, Schematic shows differential gene expression (log2(fold change)) for 3 biological replicates (donors E-G) in CPSF6 knock-out primary CD4+ T cells as compared to NT controls overlayed on schematic of signaling events downstream of the type I and type II interferon receptors, including ISGs that are induced by these signaling pathways. g, Chart shows TRIM5α band density divided by β-actin band density in knock-out validation blot for donor E (Fig 2b). Density measurements are normalized to NT control. h, Graphs show mean fluorescence intensity (MFI) of CD4 (first), CXCR4(second) CCR5 (third) and CD25 (fourth) in primary CD4 + T cells from 3 biological replicates (donors H-J) at day 5 post-editing as measured by immunostaining a [file ppat.1013745.s002.tif]

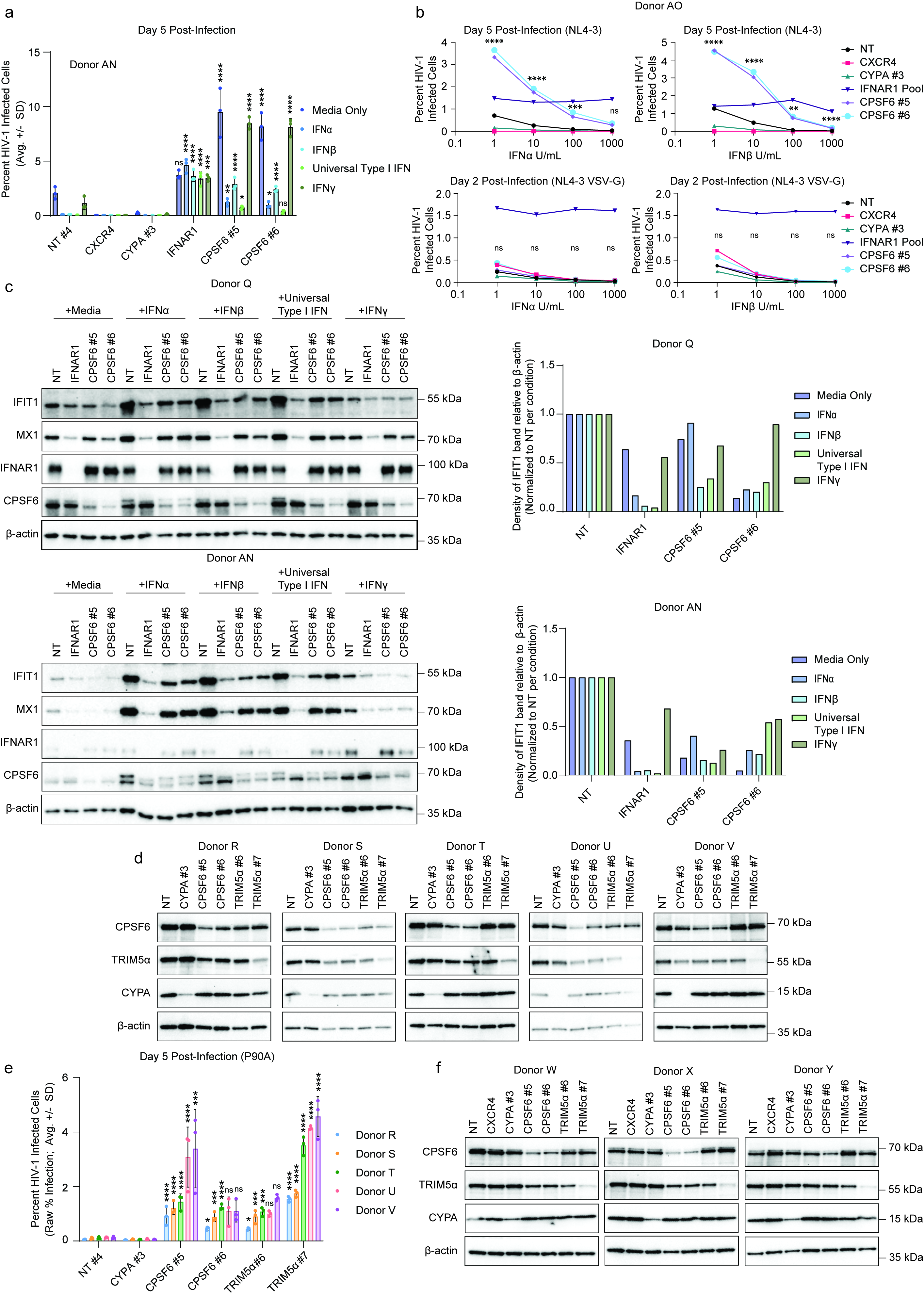

Supplement: S3 Fig — a, HIV-1 infectivity (Raw % GFP positive cells) at day 5 post-challenge with HIV-1 NL4-3 nef:IRES:GFP in indicated knock-out primary CD4+ T cells from 1 biological replicate (AN) as assessed by flow cytometry. Cells were pre-treated with 100 U/mL IFNα, IFNβ, IFNγ, Universal Type I IFN, or media only control. Each bar represents the average of technical triplicates + /- SD with individual data points shown. Statistics were calculated relative to the NT control per condition by one-way ANOVA with Dunnet’s test for multiple comparisons; * = p ≤ 0.05, ** = p ≤ 0.01, *** = p ≤ 0.001, **** = p ≤ 0.0001 b, HIV-1 infectivity (Raw % GFP positive cells) at day 5 post-challenge with HIV-1 NL4-3 nef:IRES:GFP (top) or VSV-G pseudotyped HIV-1 NL4-3nef:IRES:GFP (bottom) in indicated knock-out primary CD4+ T cells from 1 biological replicate (AO) as assessed by flow cytometry. Cells were pre-treated with indicated doses of IFNα (left) or IFNβ (right) for 16 hours prior to infection. Statistics are shown for CPSF6 #6 compared to the non-targeting (NT) control per condition, calculated by one-way ANOVA with Dunnet’s test for multiple comparisons; * = p ≤ 0.05, ** = p ≤ 0.01, *** = p ≤ 0.001, **** = p ≤ 0.0001, ns = not significant. c, Immunoblots (left) show expression of representative ISGs (IFIT1 and MX1) in knock-out primary CD4+ T cells treated with 10 U/mL IFNα, IFNβ, Universal Type I IFN, IFNγ, or media-only control for 16 hours in 2 biological replicates (donors Q and AN). Charts (right) show IFIT1 band density divided by β-actin band density in adjacent blots. Density measurements are normalized to NT controls per condition. d, Immunoblot shows knock-out of CPSF6, TRIM5α, and CYPA in primary CD4+ T cell protein lysates harvested at day 4 post-editing in 5 biological replicates (donors R-V). e, HIV-1 infectivity (Raw % GFP positive cells) at day 5 post-challenge with HIV-1 NL4-3 nef:IRES:GFP P90A capsid mutant in indicated knock-out primary CD4+ T cells from 5 biological repli [file ppat.1013745.s003.tif]

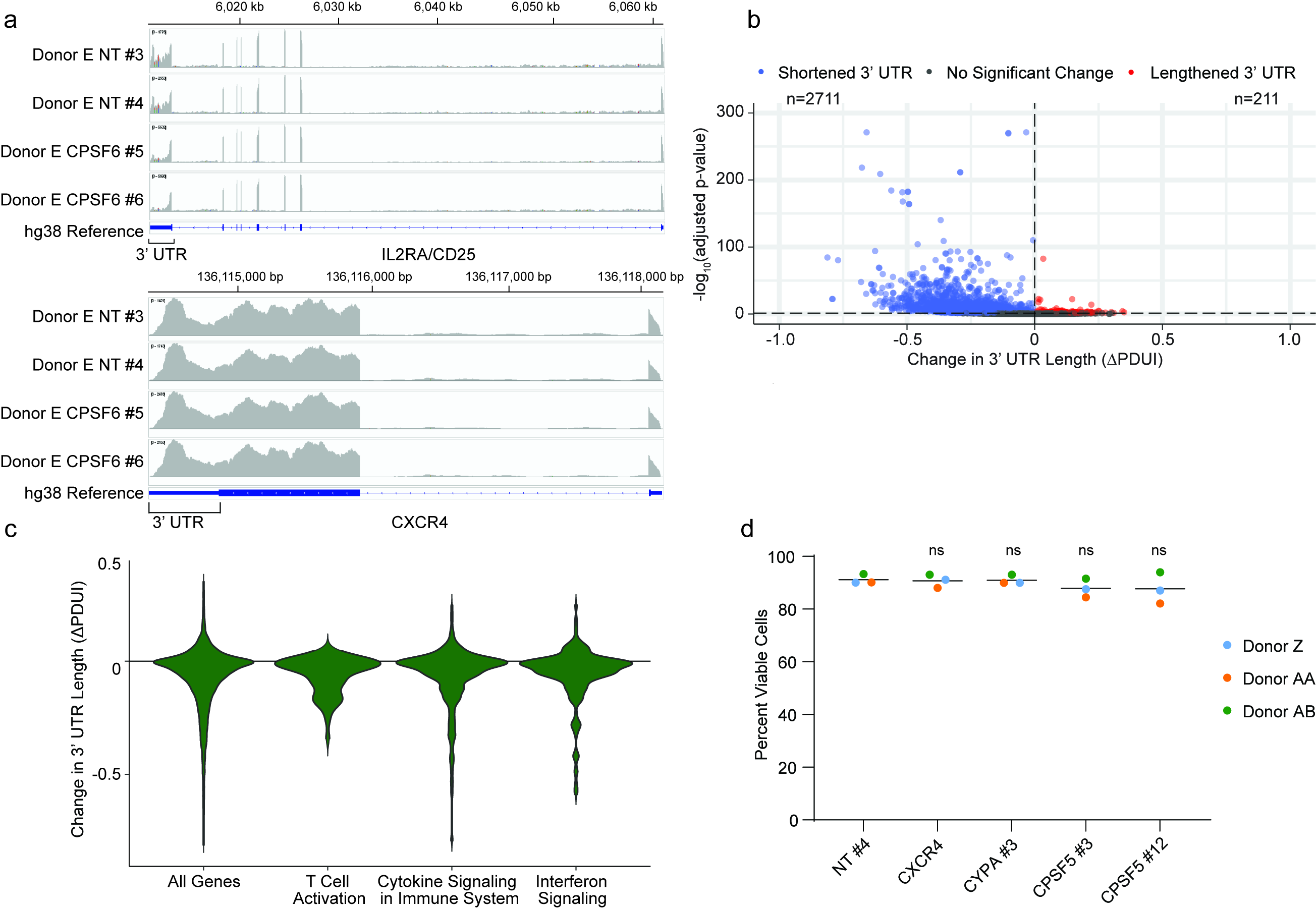

Supplement: S4 Fig — 3’ UTRs are annotated for each gene. b, Volcano plot shows DaPars analysis of changes in 3’ UTR lengths for CPSF6 knock-out versus NT control primary CD4+ T cells from 3 biological replicates (donors E-G). 3’ UTRs are designated shortened (blue) for ΔPDUI < 0 and -log10(adjusted p-value) < 0.05 and lengthened (red) for ΔPDUI > 0 and -log10(adjusted p-value) < 0.05. Dots represent tested genes. c, Violin plot shows comparison of ΔPDUI values from DaPars analysis in all tested genes as compared to genes in the indicated gene sets (GO:0042110:T Cell activation, R-HSA-1280215: Cytokine Signaling in Immune system, R-HSA-913531: Interferon Signaling) in 3 biological replicates (donors E-G). d, CPSF5 knock-out primary CD4+ T cells exhibit similar viability to NT controls at day 4 post-editing as assessed via amine dye stain and flow cytometry. Dots represent cell viability (% Ghost Red negative cells) per condition, horizontal lines represent the average of viability measurements in 3 biological replicates (donors Z-AB). (TIF) [file ppat.1013745.s004.tif]

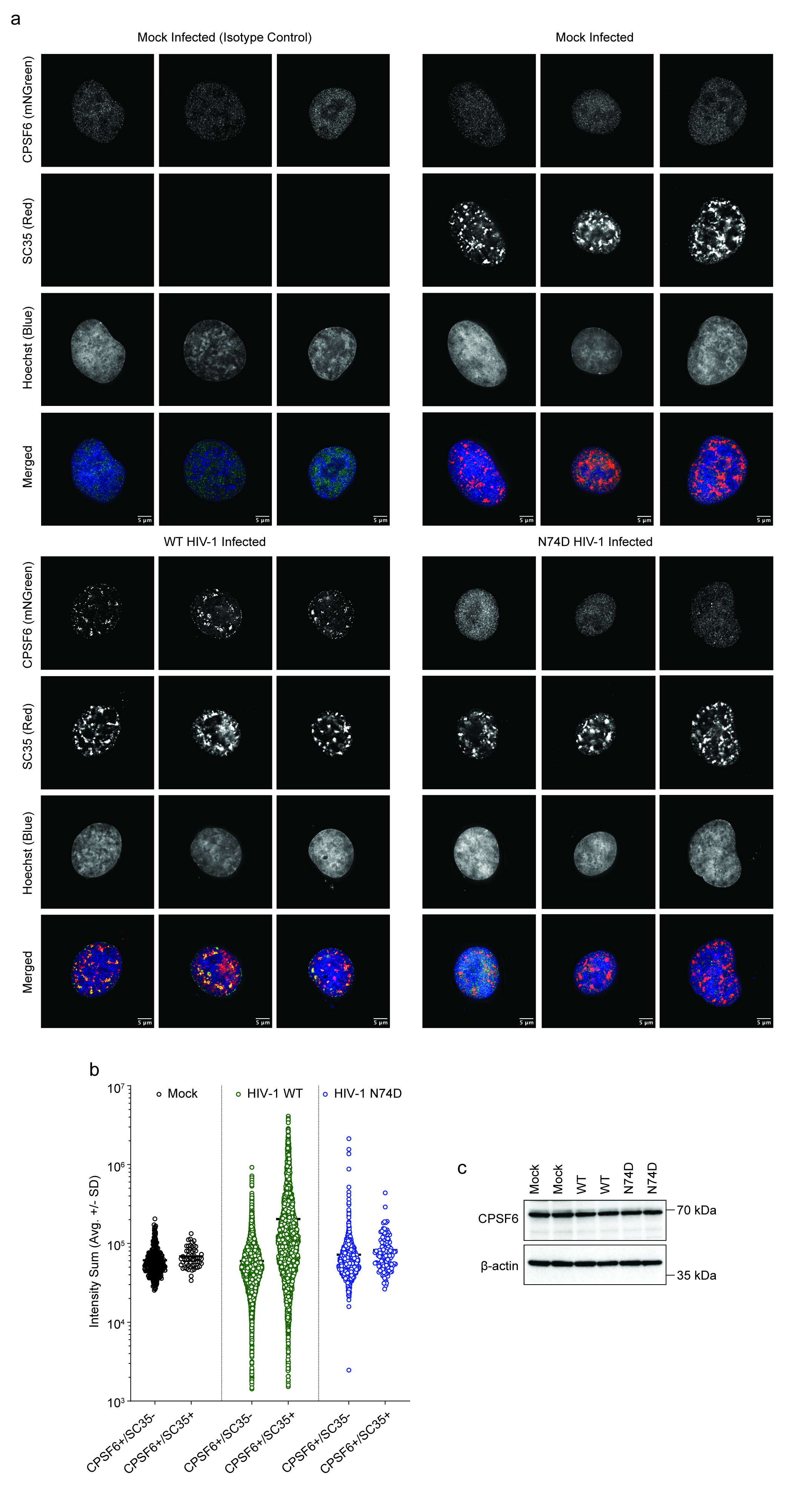

Supplement: S5 Fig — a, Widefield deconvolution fluorescent microscopy images of HT1080-CPSF6-mNGreen cells with immunofluorescent staining for a marker of nuclear speckles, SC35, or an IgG isotype control (top left set of images). Cells were mock infected (top 2 sets of images) or infected with VSV-G pseudotyped WT (bottom left set of images) or N74D capsid mutant (bottom right set of images) HIV-1-NL4-3 at an MOI of 10 at 6 hours post-infection. CPSF6-mNGreen shown in green, Hoechst DNA stain shown in blue, SC35 shown in red. 3 representative images shown per condition. b, Graph shows quantification of the intensity sum of fluorescent signal for CPSF6 + /SC35- and CPSF6 + /SC35 + puncta in HT1080-CPSF6-mNGreen cells mock infected or infected with VSV-G pseudotyped WT or N74D capsid mutant HIV-1-NL4–3 at an MOI of 10 depicted in panel a. CPSF6 puncta were detected and SC35 nuclear speckles were segmented using Imaris, and the detected CPSF6 puncta were then classified into either colocalizing or non-colocalizing with SC35. c, Immunoblot shows expression of CPSF6 in HT1080-CPSF6-mNGreen cells mock infected or infected with VSV-G pseudotyped WT or N74D capsid mutant HIV-1-NL4-3 at an MOI of 10 at 6 hours post-infection in 2 technical replicates. (TIF) [file ppat.1013745.s005.tif]

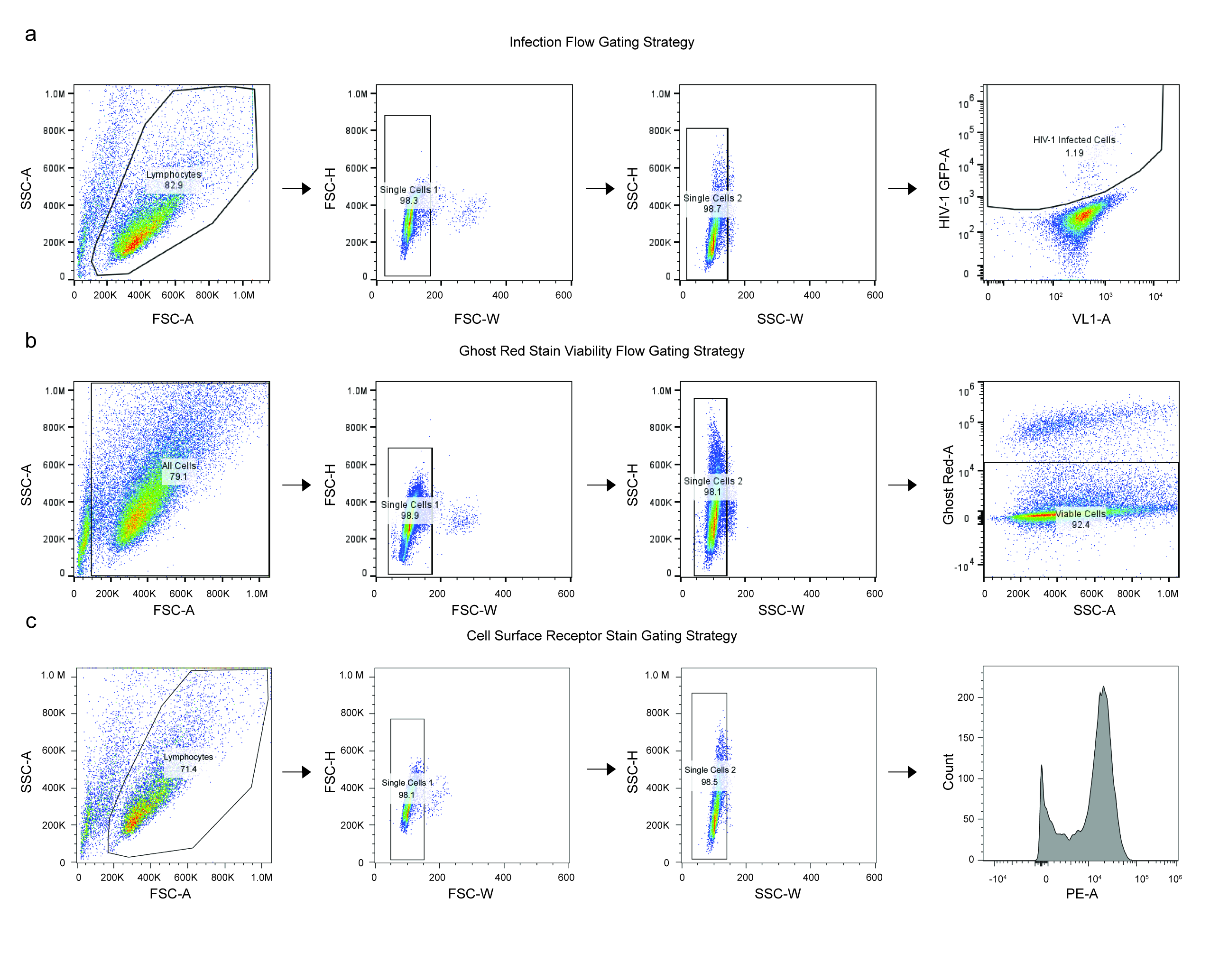

Supplement: S7 Fig — a, Flow gating strategy for quantification of percent HIV-1 NL4–3 nef:IRES:GFP infected primary CD4 + T cells through sequential application of a lymphocyte gate, two single-cell gates, and GFP autofluorescence exclusion (FlowJo v10.10). For cells sorted using the MACSQuant Tyto sorter, cells were gated on backscatter (BSB) rather than forward scatter (FSC). b, Flow gating strategy for quantification of percent viable primary CD4+ T cells through sequential application of an all-cell gate, two single-cell gates, and a fluorophore gate (FlowJo v10.10). c, Flow gating strategy for quantification of fluorescence intensity of cell surface receptors through sequential application of a lymphocyte gate, two single-cell gates, and a histogram showing cell count versus fluorescence intensity measurement of the fluorophore of interest (FlowJo v10.10). (TIF) [file ppat.1013745.s007.tif]
